# Metabolic Alterations in Myotonic Dystrophy Type 1 and Their Correlation with Lipin

**DOI:** 10.3390/ijerph18041794

**Published:** 2021-02-12

**Authors:** Tiago Mateus, Filipa Martins, Alexandra Nunes, Maria Teresa Herdeiro, Sandra Rebelo

**Affiliations:** Institute of Biomedicine (iBiMED), Department of Medical Sciences, University of Aveiro, 3810-193 Aveiro, Portugal; tiagomateus30@hotmail.com (T.M.); samartins@ua.pt (F.M.); alexandranunes@ua.pt (A.N.); teresaherdeiro@ua.pt (M.T.H.)

**Keywords:** myotonic dystrophy type 1, metabolic syndrome, dyslipidemia, lipin, insulin resistance

## Abstract

Myotonic dystrophy type 1 (DM1) is an autosomal dominant hereditary and multisystemic disease, characterized by progressive distal muscle weakness and myotonia. Despite huge efforts, the pathophysiological mechanisms underlying DM1 remain elusive. In this review, the metabolic alterations observed in patients with DM1 and their connection with lipin proteins are discussed. We start by briefly describing the epidemiology, the physiopathological and systemic features of DM1. The molecular mechanisms proposed for DM1 are explored and summarized. An overview of metabolic syndrome, dyslipidemia, and the summary of metabolic alterations observed in patients with DM1 are presented. Patients with DM1 present clinical evidence of metabolic alterations, namely increased levels of triacylglycerol and low-density lipoprotein, increased insulin and glucose levels, increased abdominal obesity, and low levels of high-density lipoprotein. These metabolic alterations may be associated with lipins, which are phosphatidate phosphatase enzymes that regulates the triacylglycerol levels, phospholipids, lipid signaling pathways, and are transcriptional co-activators. Furthermore, lipins are also important for autophagy, inflammasome activation and lipoproteins synthesis. We demonstrate the association of lipin with the metabolic alterations in patients with DM1, which supports further clinical studies and a proper exploration of lipin proteins as therapeutic targets for metabolic syndrome, which is important for controlling many diseases including DM1.

## 1. Introduction

Myotonic dystrophy type 1 (DM1) is a multisystemic and autosomal dominant hereditary disease mainly characterized by progressive distal muscle weakness and myotonia (sustained muscle contractions) [1,2,3,4,5]. DM1 is caused by the expansion of unstable repetitions of cytosine-thymine-guanine trinucleotide (CTG) in the 3’ untranslated region (3’UTR) of the *Myotonic Dystrophy Protein Kinase* (*DMPK*) gene located at chromosome 19q13.3 [4,6,7].

Metabolic syndrome (MetS) in patients with muscular disorders are significantly higher than in general population due to abnormal lipid metabolism, such as insulin resistance, hypertriglyceridemia, increased fat mass, high levels of low-density lipoprotein (LDL), low levels of high-density lipoproteins (HDL) and abdominal obesity [8,9]. These abnormalities seem to be related to lipin, since it is a key enzyme that regulates lipid metabolism and signaling, and is a transcriptional co-activator of fatty acid β-oxidation [10,11,12,13,14]. Other important features associated to the effects of lipin alterations that have been studied in the last decade and demonstrated by previous studies will be detailed below.

Therefore, the purpose of this review is to provide an overview of the metabolic changes observed in patients with DM1 and to correlate them with current knowledge of lipin and their role in DM1. We briefly summarize the DM1 epidemiology and the underlying molecular mechanism, after which we provide clinical evidence of abnormal lipid metabolism in patients with DM1. The role of lipin in DM1 is also discussed in this review.

## 2. Epidemiology and Molecular Characteristics of DM1

Muscular dystrophies are a group of inherited muscle disorders, in which one or more genes necessary for normal muscular function are mutated, resulting in progressive weakness and loss of muscle mass with a variety of severity degrees [15,16,17]. Concerning muscular dystrophies, there are nine major forms namely, Duchenne muscular dystrophy (DMD), Becker muscular dystrophy (BMD), congenital muscular dystrophy (CMD), myotonic dystrophy (MD), facioscapulohumeral muscular dystrophy (FSHD), Emery–Dreifuss muscular dystrophy (EDMD), distal muscular dystrophy (DD), limb-girdle muscular dystrophy (LGMD) and oculopharyngeal muscular dystrophy (OPMD) [15,16,17]. 

DM1 also known as Steinert’s disease, is the most common form of muscular dystrophy in adults, with a prevalence of 1 in 3000 and 8000 individuals worldwide (Table 1) [1,4,18,19]. The main clinical feature of DM1 is myotonia (sustained muscle contractions) at the skeletal muscle level, progressive weakening of the distal muscles, and also affects other organ systems (e.g., eyes, heart, lungs) (Table 1) [1,4,18]. Additionally, other multisystem characteristics are observed namely, insulin resistance, dyslipidemia, defects in cardiac conduction, gonodal atrophy, alterations in the central nervous system (CNS), and breathing problems (Table 1) [1,2,20]. DM1 is caused by the expansion of unstable repetitions of CTG in the 3’UTR *DMPK* gene (Figure S1). The latter gene gives rise through extensive alternative splicing to 6 major DMPK isoforms proteins (DMPK A, B, C, D, E, and F) in both humans and mice, and 1 isoform (DMPK G) only present in humans (Figure S1) [6,20,21,22]. The expanded CUG repeats of the DMPK protein accumulates as nuclear foci compromising nuclear functions, and the expanded mRNA products are toxic to cells, affecting the normal processing of other genes in different tissues [4,6,7,18].

The number of CTG repeats in the *DMPK* gene is polymorphic and is correlated with the severity of DM1. In healthy population it varies between 5–37 repetitions. Individuals who have the DM1 pre-mutation have at least 38–49 repetitions being considered generally asymptomatic. However, they can pass the disease to the next generation. In contrast, individuals with repeats number comprised between 50–4000 are considered patients with DM1 [5,23]. In average, CTG expansions increase an additional 200 repetitions when transmitted from one generation to the next, leading to the disease anticipation, meaning that the disease symptoms will appear earlier and with higher severity in the subsequent generation [5,23]. In addition, although DM1 is more common in early adulthood, it can also affect fetal development and postnatal growth in individuals with large number of CTG expansions. Due to DM1 clinical heterogeneity, it is routinely subdivided according to the severity of symptoms and the age of disease onset. These categories are important to provide adequate DM1 recognition, diagnosis, and prognosis. In other words, the phenotype is divided into three categories (mild, classic, and severe) and clinically categorized as congenital (<1 month and ≥1000 CTG repeat length), infantile (1 months to 10 years and >500 CTG repeat length), juvenile (>10 to 20 years and >400 CTG repeat length), adult-onset (>20 to 40 years and 150 to 1000 CTG repeat length), and late-onset (>40 years and 50 to 149 CTG repeat length) [23,24,25,26].

## 3. Physiological and Systemic Features of DM1

Besides progressive muscle weakness and myotonia, the most common features observed in patients with DM1 are endocrine and metabolic alterations, respiratory and cardiac dysfunctions (Table 1). Endocrine dysfunctions have an increased incidence in patients with DM1, especially gonadal insufficiency (hypogonadism), diabetes, and thyroid disorders [27,28]. Hypogonadism is the most frequent feature and is characterized by low serum testosterone levels being more prevalent in men (Table 1) [27]. Palpable thyroid gland abnormalities have been described in 20% of patients with DM1 [27,28]. These endocrine alterations are correlated with metabolic dysfunctions in these patients which will be detailed below.

Overall respiratory dysfunction (respiratory failure or aspiration) is the most common cause of death in patients with DM1, since respiratory disease has a complex etiology in these patients, combining both peripheral respiratory dysfunction and central respiratory drive dysfunction, as well as upper airway muscles dysfunction (obstructive sleep apnea and aspiration) and high risk of pulmonary infections [29,30]. The mortality rate involving respiratory dysfunction ranges from 51% to 76% specially in congenital form (Table 1) [29,30]. Cardiac dysfunction is the second common cause of death, about 30% of mortality in adults with DM1. Cardiac involvement in DM1 includes conduction disease, atrial and ventricular tachyarrhythmias, atrioventricular block, left-ventricular systolic dysfunction, and myocardial fibrosis resulting in sudden death (Table 1) [31,32,33].

These systemic features may be associated to *DMPK* gene alterations, given that *DMPK* mRNA is highly expressed in skeletal, cardiac, smooth muscle and in smaller amount in different areas of the brain. DMPK is highly expressed during muscle development between weeks 9–16 and is associated to myogenic differentiation, meaning that DMPK has an important role during myogenesis [6,34,35,36]. In addition to muscle development, DMPK is also involved with the maintenance of ion homeostasis (Dihydropyiridine-Voltage-Dependent L-type Calcium receptors) and sodium channels. Furthermore, DMPK also modulates the chloride channel phospholemman (PLN), the activation or inhibition of Sarco/Endoplasmic reticulum Ca^2+^-ATPase (SERCA) through phosphorylation of phospholamban and sarcolipine. As such, it is possible to state that DMPK is involved in skeletal muscle contraction and relaxation and ion homeostasis. In the case of DM1 disease, DMPK may be related to myotonia and also could be involved indirectly or directly with the cytoskeleton myosin phosphatase target subunit (MYPT1), serum response transcription factor (SRF), and the nuclear envelope protein lamin A/C [6,37,38,39].

## 4. DM1 Underlying Molecular Mechanisms

**Figure 1:**
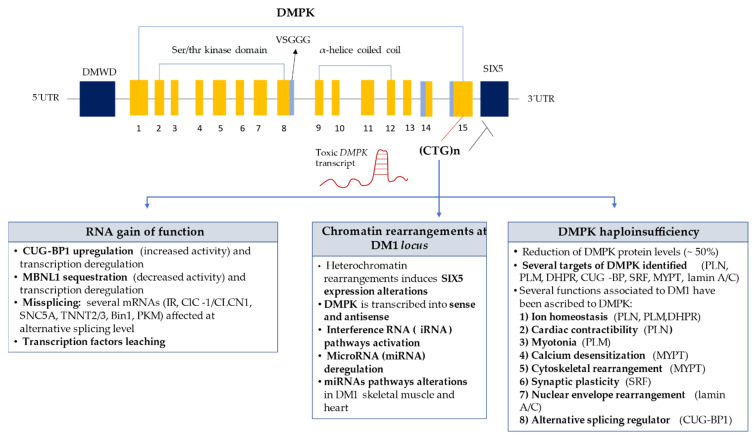
**Molecular Mechanisms in DM1.** Toxic CUG-containing transcripts forms secondary structures and accumulates in the nucleus of DM1 cells, causing multisystemic effects of DM1 throughout RNA gain-of-function mechanism, Chromatin rearrangements at the DM1 locus and DMPK haploinsufficiency [6,21,40,41,42,43,44,45,46,47,48]. CUGBP1-CUGBP-Elav-like family member 1; MBLN1—muscleblind-like protein 1; Insulin receptor (IR); DHPR—Voltage-Dependent L-type Calcium; PLM—Phospholemman, PLN—Phospholamban; SRF—Serum response factor; MYPT—Myosin phosphatase; CIC/CLCN1—Voltage-gated chloride channel; SNC5A—Sodium channel protein type 5 subunit alpha; TNNT2/3—Troponin T 2/3; Bin1—Bridging integrator 1; PKM—Pyruvate kinase.

To date the identification of the molecular mechanisms underlying this pathology is not yet fully understood. However, there are three hypotheses that are more consensual within the scientific community namely the rearrangement of DM1 locus, *DMPK* haploinsufficiency, and gain of toxic RNA function (Figure 1).

Regarding rearrangement of the DM1 locus, *DMPK* gene is transcribed into sense and anti-sense transcripts (Figure 1) [7]. Anti-sense transcripts could be involved in chromatin ultrastructure regulation, since it is known that anti-sense *DMPK* transcripts extend into an insulator element located between *DMPK* and *SIX5* genes [7,40]. The formation of double strand RNA structures due to the folding of CUG transcripts into hairpin structures, or due to complementary hybridization between sense and anti-sense *DMPK* transcripts might activate iRNA [22,39]. Furthermore, microRNA (miRNA) deregulation could be an additional mechanism involved in DM1 biogenesis, since it has been found altered in skeletal muscle and heart of patients with DM1 [41,42].

Concerning *DMPK* haploinsufficiency (Figure 1), it is also known that about ~50% of *DMPK* expression and activity are decreased due to CTG expansions (haploinsufficiency) and that DMPK is crucial for the phosphorylation of certain essential proteins for muscle contraction and relaxation, such as phospholamban (PLB), phospholemman (PLM), sarcolipine (SLN), and lipin [21,43,44]. PLB and SLN when phosphorylated interact with SERCA allowing muscle contraction, through calcium output. Due to the decrease in DMPK levels, phospholamban and sarcolipine are not phosphorylated, in turn SERCA is constantly inhibited, leading to an increase concentration of calcium in the cytoplasm, causing sustained muscle contraction (myotonia) [6,21,29,43,44].

Gain of toxic RNA function is another plausible DM1 mechanism (Figure 1). The mutant *DMPK* mRNA with the CUGexp repeats accumulates in the nucleus as a ribonuclear foci folding into ‘RNA hairpins’, sequestering and deregulating RNA-binding proteins (RBP) crucial for alternative cell processing of other genes and/or mRNAs, leading to the complexity of the DM1 phenotype. Among the RBP associated with abnormal splicing mechanism related with DM1 are muscleblind-like protein1 (MBNL1) and CUG-BP-ELAV-Like family member1 (CUGBP1 or CELF1) [43,44]. MBNL1 protein is responsible for splicing regulation of several hundreds of transcripts, regulating mRNA transport and decay. MBLN1 has high affinity to the CUG repeats of the RNA, thus being sequestered in the nucleus by the *DMPK* CUGexp mRNA, reducing its activity and availability in the cell [5,43]. CUGBP1 is linked to a “short single-stranded CUG repeat”, so it is not sequestered as MBNL1 [21]. It is hypothesized that hyperphosphorylation and stabilization of CUGBP1 occurs [21] through a PKC activation induced by a mutant mRNA through an unknown mechanism [21]. Both MBNL1 and CUGBP1 proteins may be responsible for some of the symptoms observed in patients with DM1 (Figure 1) [6,21,45,46,47,48]. Additionally, being DMPK an important protein kinase is reasonable to deduce that protein phosphorylation is also an important regulatory mechanism in DM1. Aberrant protein phosphorylation is recognized as a critical step in the pathogenesis of several diseases, such as Alzheimer’s disease and cancer [49,50,51,52].

## 5. Metabolic Dysfunction in DM1

MetS represents an accumulation of at least three metabolic abnormalities out of the five conditions, which are hypertension, central obesity, insulin resistance, atherogenic dyslipidemia, and pro-inflammatory state [53]. Metabolic syndrome can lead to cardiovascular disease, diabetes mellitus, vascular, and neurological problems such as cerebrovascular accident [9,53,54]. Metabolic abnormalities can become a syndrome if a patient presents at least three of the following metabolic risk factors:

- Waist circumference (or abdominal obesity) more than 40 inches or ≥102 cm in men and 35 inches or ≥88 cm in women [9,53,54];
- Elevated triglycerides (TAG) ≥150 milligrams per deciliter of blood (mg/dL) or higher [9,53,54];
- Reduced high-density lipoprotein cholesterol (HDL) less than <40 mg/dL (1.0 mmol/L) in men and <50 mg/dL (1.3 mmol/L) in women that is associated with increased cholesterol levels [9,53,54];
- Normal value of fasting glucose <100 mg/dL. Elevated fasting glucose of ≥l00–125 mg/dL is considered pre-diabetes and 126 mg/dL or higher is considered diabetes [9,53,54];
- Blood pressure values of systolic ≥130 mmHg or higher and/or diastolic ≥85 mmHg or higher [9,53,54].

Literature review was conducted in Web of Science and PubMed. Data were gathered and extracted to a structured table (Table S1) with authors, year of publication, number of participants (DM1 patients and controls), age and sex, CTG repeat length, body mass index (BMI) and waist circumferences, insulin metabolism, HOMA-IR, glucose metabolism, and lipid metabolism. Data regarding age, sex, CTG repeat length, BMI were collected to characterize the population. One summary table (Table 2) was created to synthesize previously reported results of abnormal metabolism studies in patients with DM1. From the included studies, all data was gathered, and upon adequate calculations they were expressed as mean ± standard deviation (SD; range) and median (Table 2). From the literature review, 18 studies evaluated metabolic alterations in patients with DM1 (summarized in Table 2), in which is it was possible to observe differences regarding lipid metabolism between patients with DM1 and control group. Clinically, insulin metabolism and HOMA-IR (Insulin resistance index) were significantly higher in patients with DM1 than control group [27,28,55,56,57,58,59,60,61,62] as demonstrated in Table 2. Glucose levels were similar in both patients with DM1 and control group [27,28,55,56,57,58,60,61,62,63,64,65,66,67]. Total cholesterol values in patients with DM1 were higher than control group [27,28,55,56,57,58,59,60,62,63,64,65,67]. LDL was also higher in patients with DM1 when compared with control group [27,28,58,59,60,62,63,64,68,69]. Whereas HDL was similar in both patients and control group [27,28,58,59,60,62,63,64,65,66,68,69]. TAG levels were higher in patients with DM1 than control group [27,28,56,57,58,59,60,62,63,64,65,66,67,68,69]. Furthermore, from the 18 studies, 11 [27,55,57,59,60,61,62,64,65,68,70] showed a significant difference (*p* ≤ 0.05) between patients with DM1 and controls (Table S1); namely, higher levels of insulin [57,59,61,62], HOMA-IR [27,57,61,65,70], total cholesterol [55,57,60,65], LDL [60,68], TAG [27,57,59,60,65,68], whereas glucose levels [64] and HDL were significantly lower [59,60,65].

Interestingly, the occurrence of MetS in patients with muscle disorders is significantly higher than in the general population [63]. Abnormal lipid metabolism is frequently observed in patients with DM1, particularly in skeletal tissue. Additionally, the main cause of this abnormality is insulin resistance (increased levels of insulin at plasma, hyperinsulinemia) and dyslipidemia (hypertriglyceridemia, low HDL cholesterolemia, high LDL cholesterolemia, and abdominal obesity) [8,9]. MetS in patients with DM1 may be associated with lipin deficiency levels. In fact, in previous studies *LPIN1* gene was reported to be among the aberrant alternative splicing genes in DM1 mouse model, particularly in the biological function of lipid metabolism, indicating that *LPIN1* gene seems to be dysregulated in DM1 [45]. Further, in a more recent study using human skeletal and heart muscle DM1-derived biopsies *LPIN1* gene was also found associated to DM1 [71]. Thus, the study of lipin is of paramount importance and the underlying mechanisms should be extensively studied. Given that, lipin could be a key to understand the metabolic features in the DM1 disease, as described in the following section.

## 6. Lipin Protein Family

**Figure 2:**
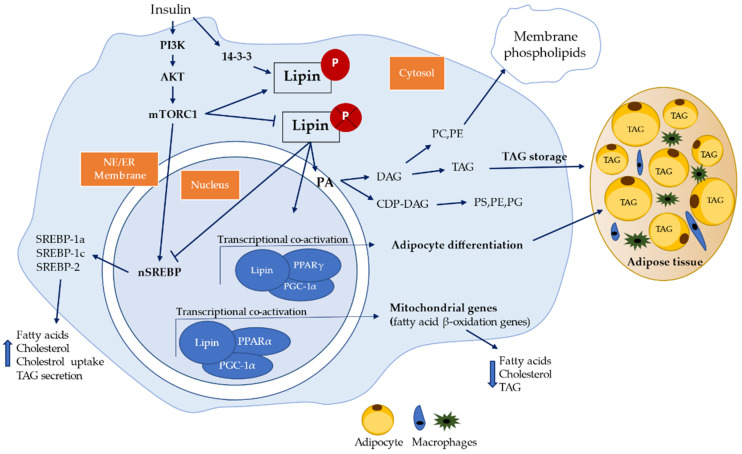
**Schematic representations of lipin pathway and signaling.** PA—phosphatidic acid; DAG—diacylglycerol; TAG—triacylglycerol; PE—phosphatidylethanolamine; PC—phosphatidylcholine; PI—phosphatidylinositol; PG—phosphatidylglycerol; CL—cardiolipin; CDP-DAG—cytidine diphosphate diacylglycerol; PS—phosphatidylserine; AKT—protein kinase B; PI3K—phosphoInositide 3-kinase; mTORC1—mitogen-activated protein kinase complex 1; nSREBP—nuclear Sterol regulatory element-binding protein; NE—nuclear envelope; ER- endoplasmic reticulum; PPARα/γ—peroxisome proliferation-activated receptor α/γ; PGC-1α—peroxisome proliferator-activated receptor gamma coactivator 1-alpha [12,72,73,76].

Lipins are phosphatidate phosphatase (PAP) enzymes that catalyze the conversion of phosphatidic acid (PA) to diacylglycerol (DAG) in TAG biosynthesis, phosphatidylethanolamine (PE) or phosphatidylcholine (PC) (Figure 2) as well as phosphatidylinositol (PI), phosphatidylglycerol (PG) and cardiolipin (CL) via cytidine diphosphate diacylglycerol (CDP-DAG) [10,11,72,73]. Phosphatidic acid is an intermediate in TAG biosynthesis that has an important function in the Kennedy pathway for the component of cell membranes and dynamic effects on intracellular and intercellular signaling pathways (Figure 2) [73].

It is also known that phosphatidic acid is the precursor of CDP-DAG used to produce phosphatidylglycerol and phosphatidylinositol, while DAG is the substrate for synthesis of other abundant phospholipids like phosphatidylcholine and phosphatidylethanolamine [73]. There are three main pathways to synthesize phosphatidic acid which are (1) de novo synthesis pathway through acylation of lysophosphatidic acid by lysophosphatidic acid acyltransferase; (2) phosphorylation of DAG; (3) hydrolysis of phospholipids by phospholipase D [74]. Some of phosphatidic acid targets are activation of NADPH, PKC-ζ, phosphatidylinositol 4-kinase, Raf proteins, phospholipase C-γ, Ras, and inhibition of protein phosphatase-1 (PP1) [75]. The effect of phosphatidic acid on Ras and Raf increases extracellular signal-related kinase activity and cell division [76]. The relative concentrations of lysophosphatidate/DAG and phosphatidic acid contributes to membrane curvature (through fusion and fission) and vesicle budding [76]. Phosphatidic acid has also been shown to activate the extracellular signal-regulated kinase (ERK), mitogen-activated protein kinase (MAPK) signaling cascades and suppressed protein kinase A (PKA) activity by a direct interaction with phosphodiesterase 4 (PDE4) and by activating mitogen-activated protein kinase (mTOR) signaling to enhance PDE activity and reduce cyclic adenosine monophosphate (cAMP) [77,78]. Additionally, phosphatidic acid can increase cell division through mTOR, and it stimulates stress fibers formation [75].

Lipin also has a transcriptional co-activator motif and can co-regulate the expression of fatty acid β-oxidation and inflammatory genes. Lipin subcellular localization is related to its dual role of transcriptional co-activator (nucleus), lipid metabolism, and lipid signaling (ER) and in cytoplasm when phosphorylated (not activated) (Figure 2) [10,11,12,13,14].

**Figure 3:**
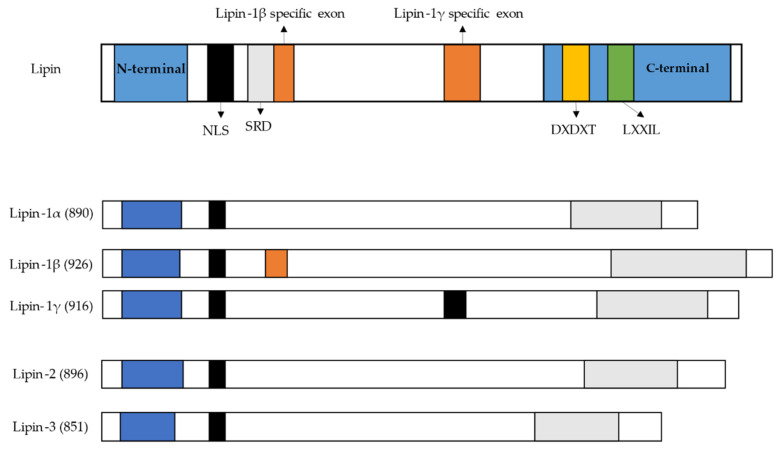
**Lipin family (lipin-1, lipin-2, and lipin-3) domains and motifs in humans.** N-terminal C-terminal NLS—nuclear localization; SRD—serine rich domain; DXDXT—phosphatidate phosphatase enzyme; LXXIL—transcriptional co-activator motif [12,72,73,76,79].

Lipin family consists of three members, which are lipin-1, lipin-2, and lipin-3. Lipin-1 was the first lipin member identified and is encoded by *LPIN1* gene [13]. Lipin-1 is a Mg^2+^ dependent protein, predominantly expressed in adipose tissue, skeletal muscle, and testis, being expressed at lower levels in other tissues such as liver, kidney, brain, heart, and lungs. There are three human lipin-1 isoforms (lipin1-α, lipin1-β, lipin1-γ) generated by alternative mRNA splicing (Figure 3) [14,72,79,80]. Lipin-1α contains 890 amino acids, predominates in pre-adipocytes during early stages of adipocytes differentiation, but most of the α-isoform is located at the nucleus as a transcriptional co-activator of lipid metabolism genes [12,13,72,79]. Lipin-1β is the longest isoform and contains an additional β-specific region of 36 amino acids (242–277) and is mainly associated with ER membrane carrying the PAP activity function. Lipin-1β predominates in mature adipocytes and induces the expression of lipogenic genes and lipid storage, whereas lipin-1γ is highly expressed in human brain compared to lipin-1α and β (Figure 3) [12,13,72,79]. Lipin-2, encoded by *LPIN2* gene, is commonly expressed in the liver, kidney, brain, lungs, macrophages, and small intestine. Lipin-3, encoded by *LPIN3* gene, has been reported that *in vivo* it cooperates with lipin-1 to influence adipose tissue PAP activity and adiposity, but it has the highest levels in small intestine, adipose tissue, and liver (Figure 3) [12,81]. All three lipin proteins are important to activate the synthesis of both major storage phospholipids, the TAG and membrane phospholipids. Lipin-1, lipin-2, and lipin-3 are also involved in many cellular process through intermediates that activate cellular signaling, including the regulation of lipid storage, lipoprotein synthesis, autophagy, inflammation, and gene expression [81]. Lipin proteins suffer post-translation modifications including phosphorylation, sumoylation, acetylation, and ubiquitination [81]. From the lipin family, only lipin-1 is regulated by insulin via mTOR phosphorylation. Dephosphorylation of lipin-1 allows to translocate freely from the cytoplasm to the nucleus and to membranes containing phosphatidic acid, such as endoplasmic reticulum, mitochondria, and autophagosomes/lysosomes (Figure 2) [81]. Lipin defects in the formation of DAG may lead to lipodystrophy or block adipocyte differentiation [14]. Also, DAG is a regulator of signaling cascades including protein kinase C (PKC) and protein kinase D (PKD) [73,82]. Activation of PKC through accumulation of DAG has been linked to insulin-resistance in obesity [73,82,83,84]. Also, activation of PKC may results in the activation of the transcription factor activator protein-1 (AP-1), which has many sequence targets in inflammation-related genes (e.g., Ciclo-oxigenase-2 (Cox2)) [85,86,87,88]. Activation of PKD cascade through DAG at the surface of autophagosomes/lysosomes, leads to fusion of autophagosomes with lysosomes to form functional autolysosomes [81]. Additionally, lipin-1 can interact with other nuclear receptors, such as PPARγ [12,89], hepatocyte nuclear factor-4α (HNF-4α) [12,90], glucocorticoid receptor (GR) [12,91], as well as non-nuclear receptor transcription factors, including nuclear factor of activated T-cells c4 (NFATc4) [92,93] and myocyte enhancer factor 2 (MEF2) [94]. Lipin-1 also represses the activity of Sterol regulatory element-binding protein 1 and 2 (SREBP1, SREBP2), and NFAT4c by inhibiting the binding of these transcription factors to their respective promoters in hepatocytes. SREBP1, SREBP2, and NFAT4c have been identified to contribute to promotion of macrophage pro-inflammatory responses and inhibition of wound healing macrophage polarization [12,14,78,81,93]. Therefore, lipin-1 can serve as gene expression activator or repressor [12].

### 6.1. Dyslipidemia and Lipin

Dyslipidemia is frequently observed in patients with DM1 [9,27,28,56,57,58,59,60,62,63,64,68,69,95]. It is a term used for lipid disorders from hyperlipidemia to hypolipidemia, which includes the elevation of plasma cholesterol, hypertriglyceridemia, low HDL, high LDL, and exceed visceral fat accumulation. It is a major risk factor for cardiovascular disease, cerebrovascular disease accident and peripheral arterial disease [9,53,54]. Dyslipidemia is strongly associated to MetS, nonalcoholic fatty liver disease (NAFLD) and is closely related with insulin resistance, since alteration of lipid metabolism and the increase of free fatty acid (FFA) flux inhibits insulin signaling leading to insulin resistance [8,9,53,56,63].

**Figure 4:**
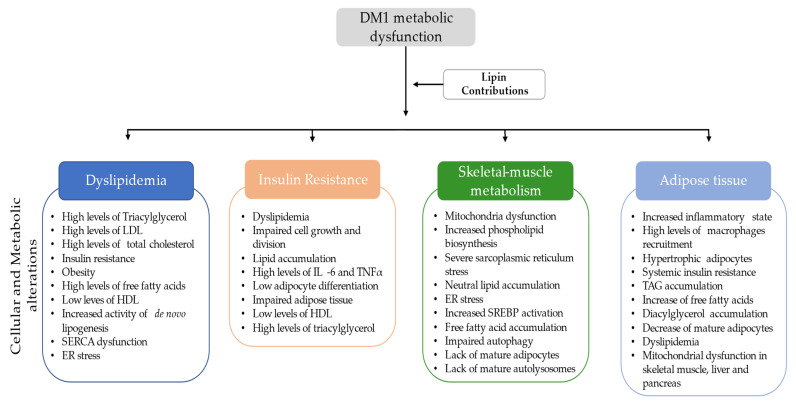
**Summary of the potential contributions of lipin dysfunction in DM1.** LDL—low-density lipoprotein; HDL—high-density lipoprotein; SERCA—sarco/endoplasmic reticulum Ca^2+^-ATPase; ER—endoplasmic reticulum; IL-6—interleukine-6; TNFα—tumour-necrosis factor-α; SREBP—Sterol regulatory element-binding protein; TAG—triacylglycerol.

These alterations in lipid metabolism frequently observed in patients with DM1 (Table 2) may be correlated with lipin metabolism, since lipin regulates adipocyte differentiation allowing TAG storage. Thus, with decreased lipin function there will be a decrease of adipocyte differentiation and an increase of TAG levels. Also, lipin has a major role in biosynthesis of phospholipids, fatty acid β-oxidation, inhibition of *de novo* lipogenesis and TAG secretion through lipoproteins. These features were also in accordance with Table 2, as with the downregulation of lipin the *de novo* lipogenesis was not inhibited and therefore there will be an increase of total cholesterol, LDL, and TAG secretion, as well as fatty acid synthesis [14,76,81,96,97,98,99,100,101]. Furthermore, the lipin deficiency may also, lead to insulin resistance, obesity, and amplification of the pro-inflammatory factors, influencing abnormal lipid metabolism, cell growth, SERCA function, endoplasmic reticulum stress, mitochondrial dysfunction, autophagosome accumulation, and lipid accumulation in skeletal muscle and liver, among other organs [8,9,14,81,96]. Therefore, it would be important to understand the correlation of lipin alteration in patients with DM1 with the high frequency of dyslipidemia and MetS. Thus, more studies with a large cohort should be taken in consideration, particularly, to evaluate the involvement of lipin with the levels of TAG, total cholesterol, HDL, LDL (Figure 4).

### 6.2. Insulin Resistance Association with Lipin

Insulin is a metabolic hormone, produced by pancreatic beta cells to regulate the glucose uptake. The major targets of insulin are adipose tissue, muscle, liver, and heart. Insulin receptor can also be found in brain, lungs, kidneys, fibroblasts, and blood cells [8]. Insulin is also a key molecule in lipid metabolism and protein synthesis, and is involved in glucose uptake in skeletal muscle and adipocytes, promotion of glycogen synthesis, inhibiting lipolysis in adipocytes, reduction of hepatic gluconeogenesis process by lipolysis inhibition, regulation of muscle proteins synthesis, and promoting cell division and growth [8,70].

Insulin is produced by the cleavage of pro-insulin. After insulin production it interacts with insulin binding receptors (IR) and insulin growth factor 1 receptor (IGF1R). Insulin receptors (IR) belong to a super family of transmembranar receptor tyrosine kinases that are encoded by the *insulin receptor (INSR)* gene located on chromosome 19p13.2 [55,70,102]. Alternative splicing of *INSR* pre-mRNA originates two isoforms, IR-A and IR-B. IR-A and IR-B are activated by auto-phosphorylation resulting in downstream cascades activation which interacts with insulin response elements 1 (IRS-1) or 2 (IRS-2), through tyrosine residues phosphorylation. IRS-1 activates the RAS/mitogen-activated protein kinase (MAPK) cascade and regulates the extracellular-signal-regulated kinase signaling (ERKS) [55,70,102,103]. Also, IRS-1 activates the phosphoInositide 3-kinase (IP3K) signaling pathway as well as protein kinase B (PKB or AKT) regulating the protein production and apoptosis via mitogen-activated protein kinase (mTOR) and glycogen synthesis via glycogen synthase kinase 3 beta (GSK-3β). Consequently, an increase of glucose transporter 4 (GLUT4) is observed enabling the glucose uptake [55,70,102,103,104]. Insulin signaling also enhances lipid storage in adipocytes, through stimulation of TAG synthesis and inhibition of lipolysis, as well as stimulation of glycogen synthesis through AKT2, glycogen synthase kinase 3 (GSK3) inhibition and glycogen synthase (GS) activation [70]. Insulin growth factor receptors (IGFR) are metabolic hormones that are structural homologs to insulin and share a common signaling pathway. There are two isoforms of IGFR namely IGF1R and IGF2R, which are produced in the liver, brain, pancreas, intestine, kidney, adipose tissue, and muscle [8].

Furthermore, it is known that insulin plays an important role in skeletal muscle growth, development, differentiation, and regeneration and in the absence of insulin stimulus, a loss in muscle mass and strength can be observed. Therefore, insulin plays a critical role controlling skeletal muscle mass through regulation of protein metabolism via two main downstream effectors, mTOR and FoxO [70]. Insulin induces the phosphorylation and activation of mTOR promoting protein synthesis, while insulin dependent phosphorylation of FoxO1 leads to a decrease in atrogenes expression [70,105], such as MuRF1 and Atrogin-1/MAFbx, thus inhibiting ubiquitin-dependent protein degradation [55,70]. The binding of insulin receptors leads to AKT/PKB activation and mTOR and FoxO phosphorylation. Phosphorylation of mTORComplex 1 allows the protein synthesis, autophagy, mitochondrial metabolism, and activation of SREBP, through lipin inhibition, which regulates *de novo* lipogenesis [8,70,73,106,107], whereas mTORComplex 2 (mTORC2) negatively regulates insulin signaling, controls cell stress response, apoptosis, and cytoskeleton organization [73]. In addition insulin stimulates the interaction between lipin-1 and 14-3-3 proteins promoting cytoplasmic retention of lipin-1 [108]. Interestingly, loss of MBNL1 function leads to changes in mTOR pathway [109] and the reduction of cyclin D3 in DM1 muscles due to abnormal increase of GSK3β switches the CUGBP1ACT to CUGBP1REP leading to a miss regulating myogenic CUGBP1 targets [110].

Insulin resistance is the inability of exogenous or endogenous insulin to increase glucose uptake and use, basically the cells stop responding to the hormone [102]. Insulin resistance is a common MetS feature observed in patients with DM1 and more than 50% of the patients have insulin resistance after dyslipidemia (Table 2) [8,63]. It is further involved in cardiovascular disease, left ventricular hypertrophy, type 2 diabetes mellitus (T2DM), atherosclerosis, hypertension, neuropathy, dyslipidemia, obesity, and loss of muscle mass and/or strength [8,70,102]. Due to high metabolic demand, insulin resistance has a significant impact in skeletal muscles, adipocytes and liver, since skeletal muscle and adipocytes account for 60–70% and 10% of insulin, respectively, for the uptake of glucose via GLUT4 [55,70,102,103]. The liver account for 30% of insulin-stimulating glucose and insulin resistance can lead to an increased triglycerides content and very-low-density-lipids (VLDL) secretion [55,70,102,103,111]. Insulin resistance also induces hyperglycemia, activation of oxidative stress (mithocondrial and endoplasmic reticulum dysfunction) and ectopic lipid accumulation [55,70].

Insulin resistance can be caused by lipid accumulation, inflammatory molecules (interleukine-6 (IL-6), tumor-necrosis factor-α (TNF-α)) secretion by adipocytes and macrophages, mitochondrial dysfunction related to fatty acid accumulation, reduced insulin mediated glucose uptake by GLUT4, and dyslipidemia (in terms of lipogenesis and lipolysis) [8,55,63,103,112]. Also, our findings summarized in Table 2 have shown an abnormal lipid metabolism in patients with DM1, which are crucial for the development of insulin resistance, such as increased levels of TAG and LDL, as well as waist circumferences, and in some cases lower levels of HDL [59,60,65]. It is recognized that lipin altered levels may lead to insulin resistance, probably through a combination of factors including impaired glucose uptake in adipose tissue and muscle, and reduced levels of adipose-derived factors, such as leptin and adiponectin (Figure 4) [113].

Interestingly, these features may be deeply involved with the lack of adipocyte stimulus and differentiation due to insulin resistance and lipin altered levels. Although, patients with DM1 have a normal glucose tolerance and a very low prevalence of diabetes, in spite of a marked insulin resistance (Table 2) [59,61], raising many questions. Thus, it is important to further study the relation and connection of lipin with insulin resistance underlying pathways, particularly, the glucose uptake and adipose tissue in these patients.

### 6.3. Skeletal Muscle Metabolism and the Lipin Role

Skeletal muscle accounts for almost half of the total body mass and energy expenditure. Additionally, it is the major site for fatty acid and glucose oxidation [114]. Lipin-1 is involved in almost all muscular PAP activity compared with lipin-2 and lipin-3 that are expressed at much lower levels in skeletal muscle. Thus, altered levels of lipin-1 affects the energy metabolism, mitochondrial enzymes [12] the glycolysis, glycogenolysis, and mitochondrial respiration, which leads to impaired skeletal muscle functions causing severe myopathies [114,115].

A previous study demonstrated a severe myopathy after deletion of lipin-1 in murine model skeletal muscle [114], and an increased phospholipid biosynthesis of PC, PE, PI, and PG, thus providing a tool for assessing lipin-1 role on skeletal muscle function and metabolism [114]. Interestingly in this study [114] the authors also found a severe sarcoplasmic reticulum (SR) stress, mitochondrial alteration, and neutral lipid accumulation of TAG, cholesterol, cholesterol esters, and increased levels of DAG, and increased SREBP activation [114].

There are three SREBP mammalian isoforms, namely, SREBP-1a, SREBP-1c, and SREBP-2 [116,117], in which, SREBP-1a regulates fatty acid and cholesterol synthesis, and cholesterol uptake [116,118,119], SREBP-1c regulates the expression of genes required for fatty acid synthesis [118,120] and is upregulated by insulin stimulation [116,119,120] and SREBP-2 regulates uptake and synthesis of cholesterol [116].

These findings are in accordance with previous studies described in Table 2, since due to deficiency of lipin-1 levels there will be an increased activation of SREBP and therefore increased fatty acids synthesis, phospholipids biosynthesis, cholesterol, and TAG levels (Figure 2) [97,98,99,100,101]. Interestingly, similar results were reported by another previous study [97] using Duchenne muscular dystrophy (DMD) mouse model, where lipin-1 knockdown lead to changes of lipid metabolism in the skeletal muscles, resulting in increased phospholipid biosynthesis and a reduction of SERCA function leading to a pronounced muscle weakness (myopathy) [97].

Previous studies also, showed mitochondria dysfunction in skeletal muscle of patients with DM1 indicating impaired oxidative skeletal muscle metabolism, low levels of ATP production and increased reactive oxygen species (ROS) production [121,122,123,124]. In addition, it was shown a 50% reduction of ATP production via mitochondrial oxidative phosphorylation system activity in patients with DM1 compared with controls [121]. As previous stated lipin-1 is a key regulator of transcriptional factors related to the fatty acid β-oxidation such as PPARα and PGC-1α promoting cellular energy consumption and the downregulation of nuclear SREBP protein, a master regulator of lipid synthesis (Figure 2) [10,14,99]. Thus, lipin-1 deficiency may be involved in mitochondrial dysfunction, which was observed in previous studies with a lipin-1 deficiency mouse model that had impaired autophagy, accumulation of dysfunctional mitochondria in skeletal muscle, and lack of mature autolysosomes formation [81,125,126,127,128]. These findings are in accordance with Table 2 data, since the lipin-1 altered levels seems to alter the mitochondrial function, and the amount of free fatty acids accumulation will increase as well as the total cholesterol and LDL synthesis through SREBP increased activity.

Interestingly one additional study has suggested a connection between lipin-1 and mitochondria through a phosphoglycerate mutase family member 5 (PGAM5), a serine/threonine specific protein phosphatase that promotes lipin-1 dephosphorylation in the nucleus and therefore, promoting the cellular energy consumption and the control of lipid metabolism through PPARα and PGC-1α. More studies are needed to understand the consequences of lipin-1 dephosphorylation in skeletal muscle mitochondria of patients with DM1 [10] as well as the true impact of lipin in skeletal muscle mitochondria function (Figure 4). 

### 6.4. Lipin Association with Adipose Tissue

Adipose tissue is mainly composed by adipocytes and acts as an energy store for skeletal muscles. There are two types of adipocytes, the white and brown adipocytes. White adipocytes store energy in a highly concentrated form, namely as TAG, mostly in a single large lipid droplets [129]. Brown adipocytes are characterized by multiple, smaller droplets of TAG, which are accessible for rapid hydrolysis and rapid oxidation of the fatty acids [129]. Adipose tissue can secret a large number of peptide hormones, adipokines and activated lipids [130], affecting energy metabolism in liver muscles and neuroendocrine pathways (behaviors related to feeding) [129]. Adipokines, for example leptin, monocyte chemoattractant protein-1/chemokine (C–C motif) ligand-2 (MCP-1/CCL2) and TNFα, modulates an inflammatory response in adipose tissue and regulate the mitochondria present in adipose tissues playing a role in the regulation of lipid and glucose metabolism [129,131].

Several studies regarding non-DM1 obese individuals have reported a relation between hypertrophic adipocytes in white adipose tissue and MetS [132,133,134]. These patients had a higher activation of inflammation, increased TAG and accumulation of lipid species, such as diacylglycerol and ceramides and systemic insulin resistance, features presented in Table 2. In addition, some of the gathered previous studies have also reported an altered adipose tissue function in patients with DM1 (Table 2) [56,57,59,64,65]. Given this findings, we hypothesized the involvement of lipin in adipose tissue dysfunction, since lipin-1α can be found in the nucleus and stimulates expression of genes involved in adipocyte differentiation such as PPARγ and CCAAT/enhancer-binding protein α [12,96]. On the other hand, lipin-1β is predominantly localized on the cytoplasm and is associated with the expression of genes involved in lipogenesis and triglyceride storage in adipocytes, including fatty acid synthase and diacylglycerol acyltransferase [96]. Given this, both lipin-1α and lipin-1β are important for normal adipogenesis and lipogenesis, as observed in previous studies with a lipin-deficient mice models which developed decreased mature adipocytes [68,125]. However, in another study the overexpression of lipin in adipose tissue of a transgenic mice caused obesity, increased lipogenic gene expression, and hypertrophic adipocytes, which could lead to improved insulin sensitivity [96,127].

Furthermore, insulin resistance and dyslipidemia in skeletal muscle are associated with inflammatory state in adipose tissue through recruiting MCP-1, TNFα, and other cytokines leading to increased free fatty acids causing a decreased TAG synthesis, accumulation of TAG and activated lipids with a long-chain of fatty acyl-CoA esters. Consequently, a disruption of mitochondrial function, and glucose transport in the skeletal muscle, liver, and pancreas is observed, thus triggering insulin resistance and dyslipidemia [9,69,129]. Interestingly, macrophages can express lipin-1, lipin-2, and lipin-3, thus lipin also has an important role in macrophage physiology and pathophysiology [135,136].

Although, we hypothesize the role of lipin altered levels in adipose tissue (Figure 4), there are evidences of adipose tissue alteration in patients with DM1, which have features of MetS summarized in Table 2. Thus, more studies regarding lipin function in adipose tissue of patients with DM1 would give insight towards these metabolic alterations and may be give rise to new underlying metabolic pathways and/or therapeutics.

Over all our findings suggests that there is indeed a correlation between lipin dysfunction and DM1 altered metabolism, particularly, a relation of lipin with dyslipidemia, insulin resistance, skeletal muscle metabolism, and adipose tissue (Figure 4). Therefore, there is a need to understand not only the underlying pathways of lipin in DM1 disease, but also, how are these pathways affected and how they potentially lead to DM1 metabolic dysregulation (Figure 4).

## 7. Future Perspectives

Of note, there are several metabolic changes observed in patients with DM1 (Table 2). These alterations are potentially involved with altered lipin expression levels and activity apparently leading to lipid metabolism abnormalities in patients with DM1. Therefore, all these aspects need further investigation. The underlying mechanisms and pathways associated to these lipid metabolic changes should be explored in DM1, since it poses interesting challenges and potential therapeutically opportunities for MetS and diabetes mellitus.

From the literature review, many questions were raised, such as: How is lipin regulated in patients with DM1? What conditions influence lipin activation and inhibition? What is the association between DMPK mRNA and/or other nuclear proteins with lipin? What are the consequences of downregulation (lipin deficiency) or overexpression in patients with DM1? Can lipin proteins be targets for the modulation of TAG storage and/or TAG secretion? These questions are in reach through studies of genetically modified mouse models, cell culture systems, transcriptome alterations, and metabolome analysis in patients with DM1 [45,71,81,127] with the aid of technology advancements and methods in measurement of lipids [107], including nuclear magnetic resonance (NMR), mass spectrometry (MS), and vibrational spectroscopy like Raman and Fourier-transform infrared (FTIR) that have the capability to analyze lipid abnormalities in a variety of biological samples [137,138,139,140,141]. In addition, there is a need to better understand and study the role of lipin in DM1, since to our current knowledge there are few studies related to this subject. Also, it would be interesting to evaluate the metabolic alterations and their relations to DM1 phenotypes (congenital-onset, infantile-onset, juvenile-onset, adult-onset, late-onset), to observe if it is possible to discriminate patients with DM1 by disease severity through metabolome analysis as a screening tool.

## 8. Concluding Remarks

In this review, we have consolidated the clinical and molecular evidence of metabolic alterations observed in patients with DM1 and the potential involvement of lipin in such metabolic alterations. In addition, evidence of altered lipin levels in DM1 mouse model and in humans has been reported [45,71], thus emphasizing the importance of this review, regarding the potential correlation between altered lipin levels and the DM1 metabolism. Also, alterations in lipidome composition have been observed in Duchenne muscular dystrophy, Becker muscular dystrophy, DM1, myotonic dystrophy type 2, among other skeletal muscle disorders [107]. These lipid alterations are important components of MetS, non-alcoholic fatty diseases, cardiovascular diseases, lipid storage disorders, vascular and neurologic disorders, diabetes mellitus, insulin resistance, atherogenic dyslipidemia, hypertension, central obesity, and pro-inflammatory state disorders [8,53,54].

Clear clinical metabolic alterations, namely, increased total cholesterol, LDL, TAG, insulin, and HOMA-IR have been demonstrated in this review between patients with DM1 and control groups. These clinical results showed not only the incidence of metabolic alterations in patients with DM1 but also, the urgent need to further study the underlying mechanisms and pathways leading to these metabolic alterations. One hypothesis is that altered levels of lipin may be associated with metabolic abnormalities found in patients with DM1, since lipin has a critical metabolic role in adipose tissue, skeletal muscle system, and liver, being localized in the cytoplasm, endoplasmic reticulum, and in the nucleus. Our findings are in accordance with previous studies [73,81,97,113,114,129,142], were alterations in lipin levels potentially led to impaired skeletal muscle functions, impaired mitochondrial function, severe sarcoplasmic reticulum stress, neutral lipid accumulation, insulin resistance, dyslipidemia, inflammatory state (macrophage) in adipose tissue, endoplasmic reticulum stress, and SREBP activation, which are common features observed in patients with DM1.

Interestingly, to our current knowledge patients with DM1 despite abnormal metabolism, rarely became glucose intolerant, and the incidence of diabetes ranges from 0 to 6.5%. Also, the blood pressure is not elevated as in patients with MetS [56,61,65,143]. Thus, patients with DM1 may be of interest for further studies on therapeutically mechanisms for diabetes and components of MetS.

Nonetheless, there is still a gap between the pathway and the mechanism underlying the molecular pathology of altered lipin levels’ downstream events regulating lipid metabolism in patients with DM1. Although, the hypothesis that altered lipin levels is a hallmark of several dysfunction in patients with DM1 is emerging. Therefore, understanding the different molecular mechanism underlying lipin dysfunction will significantly increase the knowledge of the physiological and pathological abnormal metabolism in patients with DM1.

## Figures and Tables

**Table 1:** Summary of principal clinical features of DM1 [1,2,7,15,16,19].

Global Clinical Features	DM1
Heredity	Autosomal dominant
Prevalence	1 in 3000 and 8000 individuals
Cancer	Reproductive tract (endometrial, ovarian and testicular), thyroid and colorectal
	High risk of tumour development
Anticipation	Present
Life expectancy	Reduced
Mortality	70%, Caused by cardiorespiratory complications
Appearance	Forehead balding
	Myopathic face
	Temporal wasting
	Ptosis
	Nasal/slurred speech
	Dysphagia
Age at onset	Childhood to adulthood
Congenital form	Present
Facial dysmorphism	Present in congenital form
Male hypogonadism	Present
Insulin Resistance	Present
Dyslipidemia	Present
Metabolic syndrome	Present
Thyroid deficiency	More common
Diabetes mellitus	High risk of development
Creatine kinase, liver enzymes and cholesterol	Elevated
Hypogammaglobulinemia	High incidence
Muscle weakness	Extreme at 50 years of age (distal)
Muscle Pain/myalgia	Less frequent
Clinical myotonia	Evident in early adulthood
Myotonia	Handgrip, tongue (distal)
Atrophy	Distal (early)
Type 1 fiber atrophy	Present
Calf hypertrophy	Absent
Cardiac arrhythmias	Present
Sudden death	More common
Sleep disorders	Present
Cognitive decline	Prominent in congenital form
Central nervous system problems	Present
Cataracts	Present
Respiratory failure	Present
Gastrointestinal problems	Present
Abdominal pain	Present
Constipation	Present
Gene	*DMPK*, chromosome 19q13.3, CTG expansion at 3’UTR
DMPK	Reduced
CNBP/ZNF-9	Normal
CUGBP1	Upregulated
MBNL1	Down regulated/sequestered
Ribonuclear inclusion	Present
Spliceopathy	Present
Transcription dysregulation	Present
MicroRNA dysregulation	Present
RNA translation	Present

**Table 2:** Summary of previously reported data concerning abnormal metabolism in patients with DM1.

**Patients Characteristics**	**DM1 (*n* = 623)** [27,28,55,56,57,58,59,60,61,62,63,64,65,66,67,68,69,70]	**Controls (*n* = 428)** [27,55,56,57,59,60,61,62,64,65,68,70]
**Patients per study**	44 ± 33; (8–115) [27,28,55,56,57,58,59,60,61,62,63,64,65,66,67,68,69,70]	96.8 ± 203.3; (3–734) [27,55,56,57,59,60,61,62,64,65,68,70]
**Age (years)**	41.7 ± 3.8; (34–47) [27,28,55,56,57,58,59,60,61,62,63,64,65,66,67,68,69,70]	42.35 ± 6.17; (33–54.6) [27,55,56,57,59,60,61,62,64,65,68,70]
**Sex**	379 F, 417 M [27,28,55,56,57,58,59,60,61,62,63,64,65,66,67,68,69,70]	347 F, 815 M [27,55,56,57,59,60,61,62,64,65,68,70]
**BMI (kg/m^2^)**	23.8 ± 1.75; (22.3–27.2); Median: 23.4 [27,28,55,57,58,59,61,63,64,65,66,67,70]	23 ± 0.72; (21.7–23.7); Median: 23.2 [27,55,57,59,61,64,65,70]
**Waist circumference (cm)**	95.8 ± 2.19; (94.3–97.4) Median: 93.43 [27,57,58,59]	84.9; Median: 87.5 [27,57,59]
**CTG repeat length**	555.22 ± 250.99; (355.9–973); Median: 413.75 [28,55,58,59,60,63,65,66,67,69,70]	ND
**Central Obesity (%)**	13.6 [63]	ND
**Metabolic syndrome (%)**	16.7–41.2 [58,63]	ND
**Insulin metabolism (pmol/L)**	125.4 ± 63.5; (51.38–186.11); Median: 73.5 **Male:** 127.8 ± 33.4; (104.26–151.45) **Female:** 119.8 ± 22,1; (104.16–135.48) [27,28,55,56,57,58,59,60,61,62]	69.9 ± 28.4; (45.8–86.0); Median: 44.29 **Male:** 56.25 **Female:** 63 [27,57,59,60,61,62]
**HOMA-IR**	3.03 ± 1.7; (1.9–6.4); Median: 2.25 **Male:** 4.7 **Female:** 3.7 [27,55,56,57,58,61,65,70]	1.43 ± 0.13; (1.3–1.6); Median: 1.42 [27,57,61,65]
**Glucose metabolism (mg/dL)**	91.84 ± 7.5; (82.7–108.5); Median: 91.4 **Male:** 90.5 ± 6.3; (86–95) **Female:** 95.9 ± 24.1; (78.9–113.0) [27,28,55,56,57,58,60,61,62,63,64,65,66,67]	89.65 ± 5.11; (81.6–95.6); Median: 90 **Male:** 83 **Female:** 88 [27,55,57,60,61,62,64,65]
**Total cholesterol (mg/dL)**	200.34 ± 15.88; (176–228); Median: 200 **Male:** 216.37 ± 50.02; (181.0–251.7) **Female:** 200.8 ± 13.9; (191.0–210.7) [27,28,55,56,57,58,59,60,62,63,64,65,67]	179.4 ± 24.5; (146.0–200.5); Median: 176.72 **Male:** 128 **Female:** 134 [27,55,57,59,60,62,64,65]
**HDL (mg/dL)**	52.42 ± 3.64; (48.2–58.0); Median: 51.4 **Male:** 44 **Female:** 61 [27,28,58,59,60,62,63,64,65,66,68,69]	51.4 ± 5.5; (45.7–56.7 51.82); Median: 51.82 **Male:** 67 **Female:** 55 [27,53,57,58,61,62,65,66]
**LDL (mg/dL)**	118.38 ± 14.65; (106.34–143.08); Median: 122.6 **Male:** 104 **Female:** 101 [27,28,58,59,60,62,63,64,68,69]	110.0 ± 17.7; (97.4–122.6); Median: 129.3 **Male:** 146 **Female:** 102 [27,59,60,62,64,68,69]
**TAG (mg/dL)**	184.9 ± 52.5; (108–274); Median: 172.4 **Male:** 194.3 ± 11.7; (186.0–202.6) **Female:** 134.1 ± 18.2; (121–147) [27,28,56,57,58,59,60,62,63,64,65,66,67,68,69]	115.9 ± 39.6; (77–168); Median: 95.2 **Male:** 75 **Female:** 105 [27,57,59,60,62,64,65,68,69]

Data expressed as mean ± SD (range). Abbreviations: NA—Not applicable; ND—Not Determined; F—Female; M—Male; BMI—Body Mass Index; HOMA-IR—Insulin resistance index HDL—High-density lipoprotein; LDL—Low-density lipoprotein; TAG—Triacylglycerol.

## Data Availability

Not applicable.

## Supplementary Materials

The following are available online at https://www.mdpi.com/1660-4601/18/4/1794/s1, Table S1: Summary data of altered metabolism in patients with DM1 and control groups. Figure S1: Schematic representation of DM1 gene and isoforms. (A) *DMPK* gene and major DMPK isoforms in humans and their domains and localization [5,6,7]. DMPK subcellular localization is confined to either endoplasmic reticulum (ER) or nuclear envelope (DMPK A and B), to mitochondria (DMPK C and D) or to cytoplasm (DMPK E, F, and G) [6,20,37]. UTR, untranslated region; *DMWD,* Dystrophia myotonica WD repeat-containing protein (represented as Blue); *DMPK, Myotonic Dystrophy Protein Kinase*; CTG, cytosine-thymine-guanine trinucleotide; LDR-Leucine rich Domain; PKD, Serine/threonine protein kinase domain. Alternative splicing sites of VSGGG and Tail (1, 2, 3, and 4) are represented as light blue.

## References

[1] Johnson N.E. (2019). Myotonic Muscular Dystrophies. Contin. Lifelong Learn. Neurol..

[2] Bozovic I., Peric S., Pesovic J., Bjelica B., Brkusanin M., Basta I., Bozic M., Sencanic I., Marjanovic A., Brankovic M. (2018). Myotonic Dystrophy Type 2–Data from the Serbian Registry. J. Neuromuscul. Dis..

[3] Vanacore N., Rastelli E., Antonini G., Bianchi M.L.E., Botta A., Bucci E., Casali C., Costanzi-Porrini S., Giacanelli M., Gibellini M. (2016). An Age-Standardized Prevalence Estimate and a Sex and Age Distribution of Myotonic Dystrophy Types 1 and 2 in the Rome Province, Italy. Neuroepidemiology.

[4] Rodríguez R., Hernández-Hernández O., Magaña J.J., González-Ramírez R., García-López E.S., Cisneros B. (2015). Altered nuclear structure in myotonic dystrophy type 1-derived fibroblasts. Mol. Biol. Rep..

[5] Thornton C.A. (2014). Myotonic Dystrophy. Neurol. Clin..

[6] Magaña J.J., Surez-Snchez R., Leyva-Garca N., Cisneros B., Hernndez-Hernndez O. (2012). Myotonic Dystrophy Protein Kinase: Structure, Function and Its Possible Role in the Pathogenesis of Myotonic Dystrophy Type 1. Advances in Protein Kinases.

[7] Cho D.H., Tapscott S.J. (2007). Myotonic dystrophy: Emerging mechanisms for DM1 and DM2. Biochim. Biophys. Acta Mol. Basis Dis..

[8] Nieuwenhuis S., Okkersen K., Widomska J., Blom P., ’t Hoen P.A.C., van Engelen B., Glennon J.C. (2019). Insulin Signaling as a Key Moderator in Myotonic Dystrophy Type 1. Front. Neurol..

[9] Takada H. (2018). Lipid Metabolism in Myotonic Dystrophy. Myotonic Dystrophy.

[10] Okuno H., Okuzono H., Hayase A., Kumagai F., Tanii S., Hino N., Okada Y., Tachibana K., Doi T., Ishimoto K. (2019). Lipin-1 is a novel substrate of protein phosphatase PGAM5. Biochem. Biophys. Res. Commun..

[11] Csaki L.S., Dwyer J.R., Li X., Nguyen M.H.K., Dewald J., Brindley D.N., Lusis A.J., Yoshinaga Y., de Jong P., Fong L. (2014). Lipin-1 and lipin-3 together determine adiposity in vivo. Mol. Metab..

[12] Chen Y., Rui B.-B., Tang L.-Y., Hu C.-M. (2015). Lipin Family Proteins—Key Regulators in Lipid Metabolism. Ann. Nutr. Metab..

[13] Péterfy M., Phan J., Reue K. (2005). Alternatively Spliced Lipin Isoforms Exhibit Distinct Expression Pattern, Subcellular Localization, and Role in Adipogenesis. J. Biol. Chem..

[14] Finck B.N., Gropler M.C., Chen Z., Leone T.C., Croce M.A., Harris T.E., Lawrence J.C., Kelly D.P. (2006). Lipin 1 is an inducible amplifier of the hepatic PGC-1α/PPARα regulatory pathway. Cell Metab..

[15] Bhatt J.M. (2016). The Epidemiology of Neuromuscular Diseases. Neurol. Clin..

[16] Theadom A., Rodrigues M., Roxburgh R., Balalla S., Higgins C., Bhattacharjee R., Jones K., Krishnamurthi R., Feigin V. (2014). Prevalence of Muscular Dystrophies: A Systematic Literature Review. Neuroepidemiology.

[17] LaPelusa A., Kentris M. (2020). Muscular Dystrophy. StatPearls [Internet].

[18] Esposito F., Cè E., Rampichini S., Monti E., Limonta E., Fossati B., Meola G. (2017). Electromechanical delays during a fatiguing exercise and recovery in patients with myotonic dystrophy type 1. Eur. J. Appl. Physiol..

[19] Ashizawa T., Gagnon C., Groh W.J., Gutmann L., Johnson N.E., Meola G., Moxley R., Pandya S., Rogers M.T., Simpson E. (2018). Consensus-based care recommendations for adults with myotonic dystrophy type 1. Neurol. Clin. Pract..

[20] Wansink D.G., van Herpen R.E.M.A., Coerwinkel-Driessen M.M., Groenen P.J.T.A., Hemmings B.A., Wieringa B. (2003). Alternative Splicing Controls Myotonic Dystrophy Protein Kinase Structure, Enzymatic Activity, and Subcellular Localization. Mol. Cell. Biol..

[21] Lee J.E., Cooper T.A. (2009). Pathogenic mechanisms of myotonic dystrophy. Biochem. Soc. Trans..

[22] Bush E.W., Helmke S.M., Birnbaum R.A., Perryman M.B. (2000). Myotonic Dystrophy Protein Kinase Domains Mediate Localization, Oligomerization, Novel Catalytic Activity, and Autoinhibition †. Biochemistry.

[23] De Antonio M., Dogan C., Hamroun D., Mati M., Zerrouki S., Eymard B., Katsahian S., Bassez G. (2016). Unravelling the myotonic dystrophy type 1 clinical spectrum: A systematic registry-based study with implications for disease classification. Rev. Neurol..

[24] Sansone V.A. (2016). The Dystrophic and Nondystrophic Myotonias. Contin. Lifelong Learn. Neurol..

[25] Turner C., Hilton-Jones D. (2014). Myotonic dystrophy. Curr. Opin. Neurol..

[26] Yum K., Wang E.T., Kalsotra A. (2017). Myotonic dystrophy: Disease repeat range, penetrance, age of onset, and relationship between repeat size and phenotypes. Curr. Opin. Genet. Dev..

[27] Passeri E., Bugiardini E., Sansone V.A., Pizzocaro A., Fulceri C., Valaperta R., Borgato S., Costa E., Bandera F., Ambrosi B. (2015). Gonadal failure is associated with visceral adiposity in myotonic dystrophies. Eur. J. Clin. Investig..

[28] Ben Hamou A., Espiard S., Do Cao C., Ladsous M., Loyer C., Moerman A., Boury S., Kyheng M., Dhaenens C.-M., Tiffreau V. (2019). Systematic thyroid screening in myotonic dystrophy: Link between thyroid volume and insulin resistance. Orphanet J. Rare Dis..

[29] Rossi S., Della Marca G., Ricci M., Perna A., Nicoletti T.F., Brunetti V., Meleo E., Calvello M., Petrucci A., Antonini G. (2019). Prevalence and predictor factors of respiratory impairment in a large cohort of patients with Myotonic Dystrophy type 1 (DM1): A retrospective, cross sectional study. J. Neurol. Sci..

[30] Hawkins A.M., Hawkins C.L., Abdul Razak K., Khoo T.K., Tran K., Jackson R.V. (2019). Respiratory dysfunction in myotonic dystrophy type 1: A systematic review. Neuromuscul. Disord..

[31] Hermans M.C., Faber C.G., Bekkers S.C., de Die-Smulders C.E., Gerrits M.M., Merkies I.S., Snoep G., Pinto Y.M., Schalla S. (2012). Structural and functional cardiac changes in myotonic dystrophy type 1: A cardiovascular magnetic resonance study. J. Cardiovasc. Magn. Reson..

[32] Guedes H., Moreno N., dos Santos R.P., Marques L., Seabra D., Pereira A., Andrade A., Pinto P. (2018). Importance of three-dimensional speckle tracking in the assessment of left atrial and ventricular dysfunction in patients with myotonic dystrophy type 1. Rev. Port. Cardiol..

[33] Chmielewski L., Bietenbeck M., Patrascu A., Rösch S., Sechtem U., Yilmaz A., Florian A.-R. (2019). Non-invasive evaluation of the relationship between electrical and structural cardiac abnormalities in patients with myotonic dystrophy type 1. Clin. Res. Cardiol..

[34] O’Cochlain D.F., Perez-Terzic C., Reyes S., Kane G.C., Behfar A., Hodgson D.M., Strommen J.A., Liu X.-K., van den Broek W., Wansink D.G. (2004). Transgenic overexpression of human DMPK accumulates into hypertrophic cardiomyopathy, myotonic myopathy and hypotension traits of myotonic dystrophy. Hum. Mol. Genet..

[35] Sarkar P.S., Han J., Reddy S. (2004). In situ hybridization analysis of Dmpk mRNA in adult mouse tissues. Neuromuscul. Disord..

[36] Groenen P.J.T.A. (2000). Constitutive and regulated modes of splicing produce six major myotonic dystrophy protein kinase (DMPK) isoforms with distinct properties. Hum. Mol. Genet..

[37] Harmon E.B., Harmon M.L., Larsen T.D., Yang J., Glasford J.W., Perryman M.B. (2011). Myotonic Dystrophy Protein Kinase Is Critical for Nuclear Envelope Integrity. J. Biol. Chem..

[38] Iyer D., Belaguli N., Flück M., Rowan B.G., Wei L., Weigel N.L., Booth F.W., Epstein H.F., Schwartz R.J., Balasubramanyam A. (2003). Novel Phosphorylation Target in the Serum Response Factor MADS Box Regulates α-Actin Transcription †. Biochemistry.

[39] Murányi A., Zhang R., Liu F., Hirano K., Ito M., Epstein H.F., Hartshorne D.J. (2001). Myotonic dystrophy protein kinase phosphorylates the myosin phosphatase targeting subunit and inhibits myosin phosphatase activity. FEBS Lett..

[40] Sicot G., Gourdon G., Gomes-Pereira M. (2011). Myotonic dystrophy, when simple repeats reveal complex pathogenic entities: New findings and future challenges. Hum. Mol. Genet..

[41] Perbellini R., Greco S., Sarra-Ferraris G., Cardani R., Capogrossi M.C., Meola G., Martelli F. (2011). Dysregulation and cellular mislocalization of specific miRNAs in myotonic dystrophy type 1. Neuromuscul. Disord..

[42] Rau F., Freyermuth F., Fugier C., Villemin J.-P., Fischer M.-C., Jost B., Dembele D., Gourdon G., Nicole A., Duboc D. (2011). Misregulation of miR-1 processing is associated with heart defects in myotonic dystrophy. Nat. Struct. Mol. Biol..

[43] Wheeler T.M., Krym M.C., Thornton C.A. (2007). Ribonuclear foci at the neuromuscular junction in myotonic dystrophy type 1. Neuromuscul. Disord..

[44] Ravel-Chapuis A., Bélanger G., Yadava R.S., Mahadevan M.S., DesGroseillers L., Côté J., Jasmin B.J. (2012). The RNA-binding protein Staufen1 is increased in DM1 skeletal muscle and promotes alternative pre-mRNA splicing. J. Cell Biol..

[45] Du H., Cline M.S., Osborne R.J., Tuttle D.L., Clark T.A., Donohue J.P., Hall M.P., Shiue L., Swanson M.S., Thornton C.A. (2010). Aberrant alternative splicing and extracellular matrix gene expression in mouse models of myotonic dystrophy. Nat. Struct. Mol. Biol..

[46] Ranum L.P.W., Day J.W. (2004). Myotonic Dystrophy: RNA Pathogenesis Comes into Focus. Am. J. Hum. Genet..

[47] Shin J.-Y., Hernandez-Ono A., Fedotova T., Östlund C., Lee M.J., Gibeley S.B., Liang C.-C., Dauer W.T., Ginsberg H.N., Worman H.J. (2019). Nuclear envelope–localized torsinA-LAP1 complex regulates hepatic VLDL secretion and steatosis. J. Clin. Investig..

[48] Hino S.-I., Kondo S., Sekiya H., Saito A., Kanemoto S., Murakami T., Chihara K., Aoki Y., Nakamori M., Takahashi M.P. (2007). Molecular mechanisms responsible for aberrant splicing of SERCA1 in myotonic dystrophy type 1. Hum. Mol. Genet..

[49] Rebelo S., Vieira S.I., da Cruz e Silva O.A.B., Esselmann H., Wiltfang J., da Cruz e Silva E.F. (2007). Tyr687 dependent APP endocytosis and abeta production. J. Mol. Neurosci..

[50] Vieira S.I., Rebelo S., Esselmann H., Wiltfang J., Lah J., Lane R., Small S.A., Gandy S., da Cruz e Silva E.F., da Cruz e Silva O.A. (2010). Retrieval of the Alzheimer’s amyloid precursor protein from the endosome to the TGN is S655 phosphorylation state-dependent and retromer-mediated. Mol. Neurodegener..

[51] Rebelo S., Domingues S.C., Santos M., Fardilha M., Esteves S.L.C., Vieira S.I., Vintém A.P.B., Wu W., da Cruz e Silva E.F., da Cruz e Silva O.A.B. (2013). Identification of a Novel Complex AβPP:Fe65:PP1 that Regulates AβPP Thr668 Phosphorylation Levels. J. Alzheimers Dis..

[52] Ardito F., Giuliani M., Perrone D., Troiano G., Muzio L. (2017). Lo The crucial role of protein phosphorylation in cell signaling and its use as targeted therapy (Review). Int. J. Mol. Med..

[53] Grundy S.M. (2016). Metabolic syndrome update. Trends Cardiovasc. Med..

[54] Rochlani Y., Pothineni N.V., Kovelamudi S., Mehta J.L. (2017). Metabolic syndrome: Pathophysiology, management, and modulation by natural compounds. Ther. Adv. Cardiovasc. Dis..

[55] Renna L.V., Bosè F., Iachettini S., Fossati B., Saraceno L., Milani V., Colombo R., Meola G., Cardani R. (2017). Receptor and post-receptor abnormalities contribute to insulin resistance in myotonic dystrophy type 1 and type 2 skeletal muscle. PLoS ONE.

[56] Rakocevic Stojanovic V., Peric S., Lavrnic D., Popovic S., Ille T., Stevic Z., Basta I., Apostolski S. (2010). Leptin and the metabolic syndrome in patients with myotonic dystrophy type 1. Acta Neurol. Scand..

[57] Daniele A., De Rosa A., De Cristofaro M., Monaco M.L., Masullo M., Porcile C., Capasso M., Tedeschi G., Oriani G., Di Costanzo A. (2011). Decreased concentration of adiponectin together with a selective reduction of its high molecular weight oligomers is involved in metabolic complications of myotonic dystrophy type 1. Eur. J. Endocrinol..

[58] Shieh K., Gilchrist J.M., Promrat K. (2010). Frequency and predictors of nonalcoholic fatty liver disease in myotonic dystrophy. Muscle Nerve.

[59] Johansson A., Boman K., Cederquist K., Forsberg H., Olsson T. (2001). Increased levels of tPA antigen and tPA/PAI-1 complex in myotonic dystrophy. J. Intern. Med..

[60] Spaziani M., Semeraro A., Bucci E., Rossi F., Garibaldi M., Papassifachis M.A., Pozza C., Anzuini A., Lenzi A., Antonini G. (2020). Hormonal and metabolic gender differences in a cohort of myotonic dystrophy type 1 subjects: A retrospective, case–control study. J. Endocrinol. Investig..

[61] Johansson A., Olsson T., Cederquist K., Forsberg H., Holst J., Ahren B. (2002). Abnormal release of incretins and cortisol after oral glucose in subjects with insulin-resistant myotonic dystrophy. Eur. J. Endocrinol..

[62] Hudson A.J., Huff M.W., Wright C.G., Silver M.M., Lo T.C.Y., Banerjee D. (1987). The role of insulin resistance in the pathogenesis of myotonic muscular dystrophy. Brain.

[63] Vujnic M., Peric S., Popovic S., Raseta N., Ralic V., Dobricic V., Novakovic I., Rakocevic-Stojanovic V. (2015). Metabolic syndrome in patients with myotonic dystrophy type 1. Muscle Nerve.

[64] Perseghin G., Comola M., Scifo P., Benedini S., De Cobelli F., Lanzi R., Costantino F., Lattuada G., Battezzati A., Del Maschio A. (2004). Postabsorptive and insulin-stimulated energy and protein metabolism in patients with myotonic dystrophy type 1. Am. J. Clin. Nutr..

[65] Matsumura T., Iwahashi H., Funahashi T., Takahashi M.P., Saito T., Yasui K., Saito T., Iyama A., Toyooka K., Fujimura H. (2009). A cross-sectional study for glucose intolerance of myotonic dystrophy. J. Neurol. Sci..

[66] Heatwole C.R., Eichinger K.J., Friedman D.I., Hilbert J.E., Jackson C.E., Logigian E.L., Martens W.B., McDermott M.P., Pandya S.K., Quinn C. (2011). Open-Label Trial of Recombinant Human Insulin-like Growth Factor 1/Recombinant Human Insulin-like Growth Factor Binding Protein 3 in Myotonic Dystrophy Type 1. Arch. Neurol..

[67] Perna A., Maccora D., Rossi S., Nicoletti T.F., Zocco M.A., Riso V., Modoni A., Petrucci A., Valenza V., Grieco A. (2020). High Prevalence and Gender-Related Differences of Gastrointestinal Manifestations in a Cohort of DM1 Patients: A Perspective, Cross-Sectional Study. Front. Neurol..

[68] Moorjani S., Gaudet D., Laberge C., Thibault M.C., Mathieu J., Morissette J., Lupien P.J., Brun D., Gagné C. (1989). Hypertriglyceridemia and Lower LDL Cholesterol Concentration in Relation to Apolipoprotein E Phenotypes in Myotonic Dystrophy. Can. J. Neurol. Sci. J. Can. Sci. Neurol..

[69] Takada H., Kon S., Oyama Y., Kimura T., Nagahata F. (2016). Liver functional impairment in myotonic dystrophy type 1. Neuromuscul. Disord..

[70] Renna L.V., Bosè F., Brigonzi E., Fossati B., Meola G., Cardani R. (2019). Aberrant insulin receptor expression is associated with insulin resistance and skeletal muscle atrophy in myotonic dystrophies. PLoS ONE.

[71] Wang E.T., Treacy D., Eichinger K., Struck A., Estabrook J., Olafson H., Wang T.T., Bhatt K., Westbrook T., Sedehizadeh S. (2019). Transcriptome alterations in myotonic dystrophy skeletal muscle and heart. Hum. Mol. Genet..

[72] Han G.-S., Carman G.M. (2010). Characterization of the Human LPIN1 -encoded Phosphatidate Phosphatase Isoforms. J. Biol. Chem..

[73] Lutkewitte A.J., Finck B.N. (2020). Regulation of Signaling and Metabolism by Lipin-mediated Phosphatidic Acid Phosphohydrolase Activity. Biomolecules.

[74] Zhukovsky M.A., Filograna A., Luini A., Corda D., Valente C. (2019). Phosphatidic acid in membrane rearrangements. FEBS Lett..

[75] Péterfy M., Phan J., Xu P., Reue K. (2001). Lipodystrophy in the fld mouse results from mutation of a new gene encoding a nuclear protein, lipin. Nat. Genet..

[76] Reue K., Brindley D.N. (2008). Thematic Review Series: Glycerolipids. Multiple roles for lipins/phosphatidate phosphatase enzymes in lipid metabolism. J. Lipid Res..

[77] Nadra K., Charles A.-S.d.P., Medard J.-J., Hendriks W.T., Han G.-S., Gres S., Carman G.M., Saulnier-Blache J.-S., Verheijen M.H.G., Chrast R. (2008). Phosphatidic acid mediates demyelination in Lpin1 mutant mice. Genes Dev..

[78] Mitra M.S., Chen Z., Ren H., Harris T.E., Chambers K.T., Hall A.M., Nadra K., Klein S., Chrast R., Su X. (2013). Mice with an adipocyte-specific lipin 1 separation-of-function allele reveal unexpected roles for phosphatidic acid in metabolic regulation. Proc. Natl. Acad. Sci. USA.

[79] Wang H., Zhang J., Qiu W., Han G.-S., Carman G.M., Adeli K. (2011). Lipin-1γ isoform is a novel lipid droplet-associated protein highly expressed in the brain. FEBS Lett..

[80] Reue K., Dwyer J.R. (2009). Lipin proteins and metabolic homeostasis. J. Lipid Res..

[81] Reue K., Wang H. (2019). Mammalian lipin phosphatidic acid phosphatases in lipid synthesis and beyond: Metabolic and inflammatory disorders. J. Lipid Res..

[82] Ter Horst K.W., Gilijamse P.W., Versteeg R.I., Ackermans M.T., Nederveen A.J., la Fleur S.E., Romijn J.A., Nieuwdorp M., Zhang D., Samuel V.T. (2017). Hepatic Diacylglycerol-Associated Protein Kinase Cε Translocation Links Hepatic Steatosis to Hepatic Insulin Resistance in Humans. Cell Rep..

[83] Ryu D., Oh K.-J., Jo H.-Y., Hedrick S., Kim Y.-N., Hwang Y.-J., Park T.-S., Han J.-S., Choi C.S., Montminy M. (2009). TORC2 Regulates Hepatic Insulin Signaling via a Mammalian Phosphatidic Acid Phosphatase, LIPIN1. Cell Metab..

[84] Chae M., Jung J.-Y., Bae I.-H., Kim H.-J., Lee T.R., Shin D.W. (2016). Lipin-1 expression is critical for keratinocyte differentiation. J. Lipid Res..

[85] Meana C., Peña L., Lordén G., Esquinas E., Guijas C., Valdearcos M., Balsinde J., Balboa M.A. (2014). Lipin-1 Integrates Lipid Synthesis with Proinflammatory Responses during TLR Activation in Macrophages. J. Immunol..

[86] Grkovich A., Armando A., Quehenberger O., Dennis E.A. (2009). TLR-4 mediated group IVA phospholipase A2 activation is phosphatidic acid phosphohydrolase 1 and protein kinase C dependent. Biochim. Biophys. Acta Mol. Cell Biol. Lipids.

[87] Grkovich A., Johnson C.A., Buczynski M.W., Dennis E.A. (2006). Lipopolysaccharide-induced Cyclooxygenase-2 Expression in Human U937 Macrophages Is Phosphatidic Acid Phosphohydrolase-1-dependent. J. Biol. Chem..

[88] Valdearcos M., Esquinas E., Meana C., Gil-de-Gómez L., Guijas C., Balsinde J., Balboa M.A. (2011). Subcellular Localization and Role of Lipin-1 in Human Macrophages. J. Immunol..

[89] Koh Y.-K., Lee M.-Y., Kim J.-W., Kim M., Moon J.-S., Lee Y.-J., Ahn Y.-H., Kim K.-S. (2008). Lipin1 Is a Key Factor for the Maturation and Maintenance of Adipocytes in the Regulatory Network with CCAAT/Enhancer-binding Protein α and Peroxisome Proliferator-activated Receptor γ 2. J. Biol. Chem..

[90] Chen Z., Gropler M.C., Mitra M.S., Finck B.N. (2012). Complex Interplay between the Lipin 1 and the Hepatocyte Nuclear Factor 4 α (HNF4α) Pathways to Regulate Liver Lipid Metabolism. PLoS ONE.

[91] Manmontri B., Sariahmetoglu M., Donkor J., Khalil M.B., Sundaram M., Yao Z., Reue K., Lehner R., Brindley D.N. (2008). Glucocorticoids and cyclic AMP selectively increase hepatic lipin-1 expression, and insulin acts antagonistically. J. Lipid Res..

[92] Kim H.B., Kumar A., Wang L., Liu G.-H., Keller S.R., Lawrence J.C., Finck B.N., Harris T.E. (2010). Lipin 1 Represses NFATc4 Transcriptional Activity in Adipocytes To Inhibit Secretion of Inflammatory Factors. Mol. Cell. Biol..

[93] Chandran S., Schilke R.M., Blackburn C.M.R., Yurochko A., Mirza R., Scott R.S., Finck B.N., Woolard M.D. (2020). Lipin-1 Contributes to IL-4 Mediated Macrophage Polarization. Front. Immunol..

[94] Liu G.-H., Gerace L. (2009). Sumoylation Regulates Nuclear Localization of Lipin-1α in Neuronal Cells. PLoS ONE.

[95] Terracciano C., Rastelli E., Morello M., Celi M., Bucci E., Antonini G., Porzio O., Tarantino U., Zenobi R., Massa R. (2013). Vitamin D deficiency in myotonic dystrophy type 1. J. Neurol..

[96] Yao-Borengasser A., Rasouli N., Varma V., Miles L.M., Phanavanh B., Starks T.N., Phan J., Spencer H.J., McGehee R.E., Reue K. (2006). Lipin Expression Is Attenuated in Adipose Tissue of Insulin-Resistant Human Subjects and Increases With Peroxisome Proliferator-Activated Receptor Activation. Diabetes.

[97] Paran C.W., Zou K., Ferrara P.J., Song H., Turk J., Funai K. (2015). Lipogenesis mitigates dysregulated sarcoplasmic reticulum calcium uptake in muscular dystrophy. Biochim. Biophys. Acta Mol. Cell Biol. Lipids.

[98] Lee J., Ridgway N.D. (2020). Substrate channeling in the glycerol-3-phosphate pathway regulates the synthesis, storage and secretion of glycerolipids. Biochim. Biophys. Acta Mol. Cell Biol. Lipids.

[99] Peterson T.R., Sengupta S.S., Harris T.E., Carmack A.E., Kang S.A., Balderas E., Guertin D.A., Madden K.L., Carpenter A.E., Finck B.N. (2011). mTOR Complex 1 Regulates Lipin 1 Localization to Control the SREBP Pathway. Cell.

[100] Grigoraş A., Amalinei C., Balan R.A., Giuşcă S.E., Avădănei E.R., Lozneanu L., Căruntu I.-D. (2018). Adipocytes spectrum—From homeostasia to obesity and its associated pathology. Ann. Anat. Anat. Anzeiger.

[101] Ferré P., Foufelle F. (2010). Hepatic steatosis: A role for de novo lipogenesis and the transcription factor SREBP-1c. Diabetes, Obes. Metab..

[102] Ormazabal V., Nair S., Elfeky O., Aguayo C., Salomon C., Zuñiga F.A. (2018). Association between insulin resistance and the development of cardiovascular disease. Cardiovasc. Diabetol..

[103] Sesti G. (2006). Pathophysiology of insulin resistance. Best Pract. Res. Clin. Endocrinol. Metab..

[104] Samson S.L., Garber A.J. (2014). Metabolic Syndrome. Endocrinol. Metab. Clin. North Am..

[105] Taillandier D., Polge C. (2019). Skeletal muscle atrogenes: From rodent models to human pathologies. Biochimie.

[106] Huffman T.A., Mothe-Satney I., Lawrence J.C. (2002). Insulin-stimulated phosphorylation of lipin mediated by the mammalian target of rapamycin. Proc. Natl. Acad. Sci. USA.

[107] Saini-Chohan H.K., Mitchell R.W., Vaz F.M., Zelinski T., Hatch G.M. (2012). Delineating the role of alterations in lipid metabolism to the pathogenesis of inherited skeletal and cardiac muscle disorders. J. Lipid Res..

[108] Péterfy M., Harris T.E., Fujita N., Reue K. (2010). Insulin-stimulated Interaction with 14-3-3 Promotes Cytoplasmic Localization of Lipin-1 in Adipocytes. J. Biol. Chem..

[109] Song K.-Y., Guo X.-M., Wang H.-Q., Zhang L., Huang S.-Y., Huo Y.-C., Zhang G., Feng J.-Z., Zhang R.-R., Ma Y. (2020). MBNL1 reverses the proliferation defect of skeletal muscle satellite cells in myotonic dystrophy type 1 by inhibiting autophagy via the mTOR pathway. Cell Death Dis..

[110] Wang M., Weng W.-C., Stock L., Lindquist D., Martinez A., Gourdon G., Timchenko N., Snape M., Timchenko L. (2019). Correction of Glycogen Synthase Kinase 3β in Myotonic Dystrophy 1 Reduces the Mutant RNA and Improves Postnatal Survival of DMSXL Mice. Mol. Cell. Biol..

[111] Wilcox G. (2005). Insulin and insulin resistance. Clin. Biochem. Rev..

[112] van Harmelen V., Rydén M., Sjölin E., Hoffstedt J. (2007). A role of lipin in human obesity and insulin resistance: Relation to adipocyte glucose transport and GLUT4 expression. J. Lipid Res..

[113] Reue K., Donkor J. (2006). Lipin: A determinant of adiposity, insulin sensitivity and energy balance. Future Lipidol..

[114] Rashid T., Nemazanyy I., Paolini C., Tatsuta T., Crespin P., Villeneuve D., Brodesser S., Benit P., Rustin P., Baraibar M.A. (2019). Lipin1 deficiency causes sarcoplasmic reticulum stress and chaperone-responsive myopathy. EMBO J..

[115] Chan E.K., Kornberg A.J., Ryan M.M. (2015). A diagnostic approach to recurrent myalgia and rhabdomyolysis in children. Arch. Dis. Child..

[116] Cheng X., Li J., Guo D. (2018). SCAP/SREBPs are Central Players in Lipid Metabolism and Novel Metabolic Targets in Cancer Therapy. Curr. Top. Med. Chem..

[117] Guo D., Bell E., Mischel P., Chakravarti A. (2014). Targeting SREBP-1-driven Lipid Metabolism to Treat Cancer. Curr. Pharm. Des..

[118] Hughes A.L., Todd B.L., Espenshade P.J. (2005). SREBP Pathway Responds to Sterols and Functions as an Oxygen Sensor in Fission Yeast. Cell.

[119] Im S.-S., Yousef L., Blaschitz C., Liu J.Z., Edwards R.A., Young S.G., Raffatellu M., Osborne T.F. (2011). Linking Lipid Metabolism to the Innate Immune Response in Macrophages through Sterol Regulatory Element Binding Protein-1a. Cell Metab..

[120] Shimano H., Shimomura I., Hammer R.E., Herz J., Goldstein J.L., Brown M.S., Horton J.D. (1997). Elevated levels of SREBP-2 and cholesterol synthesis in livers of mice homozygous for a targeted disruption of the SREBP-1 gene. J. Clin. Investig..

[121] García-Puga M., Saenz-Antoñanzas A., Fernández-Torrón R., de Munain A.L., Matheu A. (2020). Myotonic Dystrophy type 1 cells display impaired metabolism and mitochondrial dysfunction that are reversed by metformin. Aging (Albany NY).

[122] Gramegna L.L., Giannoccaro M.P., Manners D.N., Testa C., Zanigni S., Evangelisti S., Bianchini C., Oppi F., Poda R., Avoni P. (2018). Mitochondrial dysfunction in myotonic dystrophy type 1. Neuromuscul. Disord..

[123] Barnes P. (1997). Skeletal muscle metabolism in myotonic dystrophy A 31P magnetic resonance spectroscopy study. Brain.

[124] Ihara Y., Mori A., Hayabara T., Namba R., Nobukuni K., Sato K., Miyata S., Edamatsu R., Liu J., Kawai M. (1995). Free radicals, lipid peroxides and antioxidants in blood of patients with myotonic dystrophy. J. Neurol..

[125] Zhang P., Reue K. (2017). Lipin proteins and glycerolipid metabolism: Roles at the ER membrane and beyond. Biochim. Biophys. Acta Biomembr..

[126] Higashida K., Higuchi M., Terada S. (2008). Potential role of lipin-1 in exercise-induced mitochondrial biogenesis. Biochem. Biophys. Res. Commun..

[127] Phan J., Reue K. (2005). Lipin, a lipodystrophy and obesity gene. Cell Metab..

[128] Phan J., Péterfy M., Reue K. (2004). Lipin Expression Preceding Peroxisome Proliferator-activated Receptor-γ Is Critical for Adipogenesis in Vivo and in Vitro. J. Biol. Chem..

[129] Guilherme A., Virbasius J.V., Puri V., Czech M.P. (2008). Adipocyte dysfunctions linking obesity to insulin resistance and type 2 diabetes. Nat. Rev. Mol. Cell Biol..

[130] Rajala M.W., Scherer P.E. (2003). Minireview: The Adipocyte—At the Crossroads of Energy Homeostasis, Inflammation, and Atherosclerosis. Endocrinology.

[131] Sartipy P., Loskutoff D.J. (2003). Monocyte chemoattractant protein 1 in obesity and insulin resistance. Proc. Natl. Acad. Sci. USA.

[132] Hepler C., Gupta R.K. (2017). The expanding problem of adipose depot remodeling and postnatal adipocyte progenitor recruitment. Mol. Cell. Endocrinol..

[133] Gustafson B., Hedjazifar S., Gogg S., Hammarstedt A., Smith U. (2015). Insulin resistance and impaired adipogenesis. Trends Endocrinol. Metab..

[134] Klöting N., Blüher M. (2014). Adipocyte dysfunction, inflammation and metabolic syndrome. Rev. Endocr. Metab. Disord..

[135] Valdearcos M., Esquinas E., Meana C., Peña L., Gil-de-Gómez L., Balsinde J., Balboa M.A. (2012). Lipin-2 Reduces Proinflammatory Signaling Induced by Saturated Fatty Acids in Macrophages. J. Biol. Chem..

[136] Balboa M.A., de Pablo N., Meana C., Balsinde J. (2019). The role of lipins in innate immunity and inflammation. Biochim. Biophys. Acta Mol. Cell Biol. Lipids.

[137] Cacciatore S., Loda M. (2015). Innovation in metabolomics to improve personalized healthcare. Ann. N. Y. Acad. Sci..

[138] Ellis D.I., Dunn W.B., Griffin J.L., Allwood J.W., Goodacre R. (2007). Metabolic fingerprinting as a diagnostic tool. Pharmacogenomics.

[139] Felgueiras J., Silva J.V., Nunes A., Fernandes I., Patrício A., Maia N., Pelech S., Fardilha M. (2020). Investigation of spectroscopic and proteomic alterations underlying prostate carcinogenesis. J. Proteom..

[140] Santos F., Magalhaes S., Henriques M.C., Fardilha M., Nunes A. (2018). Spectroscopic Features of Cancer Cells: FTIR Spectroscopy as a Tool for Early Diagnosis. Curr. Metab..

[141] Santos F., Magalhães S., Henriques M.C., Silva B., Valença I., Ribeiro D., Fardilha M., Nunes A. (2019). Understanding Prostate Cancer Cells Metabolome: A Spectroscopic Approach. Curr. Metab..

[142] Schweitzer G.G., Collier S.L., Chen Z., Mccommis K.S., Pittman S.K., Yoshino J., Matkovich S.J., Hsu F.-F., Chrast R., Eaton J.M. (2019). Loss of lipin 1-mediated phosphatidic acid phosphohydrolase activity in muscle leads to skeletal myopathy in mice. FASEB J..

[143] Peric S., Stojanovic V.R., Nikolic A., Kacar A., Basta I., Pavlovic S., Lavrnic D. (2013). Peripheral neuropathy in patients with myotonic dystrophy type 1. Neurol. Res..

