# Supplementary material for: Metabolic Alterations in Myotonic Dystrophy Type 1 and Their Correlation with Lipin
Source: Int J Environ Res Public Health. 2021 Feb 12;18(4):1794. doi: 10.3390/ijerph18041794 (PMC7918590; doi:10.3390/ijerph18041794)
Supplement: Supplementary file 1 [file ijerph-18-01794-s001.pdf]

Table S1. Summary data of altered metabolism in patients with DM1 and controls.

Author, , Country, Year	Sample	CTG repeat length, BMI and Waist circumference	Insulin metabolism (pmol/L)	HOMA-IR	Glucose metabolism (mg/dL)	Lipid metabolism (mg/dL)
Passeri, E., et al. [27], Italy, 2015	**DM1 (n):** 31<br>Sex: 31M<br>Age (Median): 45y<br>**Control** (n): 32<br>Sex: 32M<br>Age (Median): 46y	Median (interquartile range):<br>CTG=ND<br>BMI (kg/m²)<br>**DM1=**24.3(22.2-27.7)<br>**Controls=**25.5(24.1-28.0); p=Ns<br>Waist circumference (cm)<br>**DM1=**96(87–103) vs **Controls=**91(87–99); p=Ns	**DM1=**71.52±103.47 vs **Controls** (median (IQR))=39.58(29.86-62.50); p= Ns	**DM1=**2.03±2.7 vs **Controls=** 1.48±1.1; p=Ns	Median (interquartile range):<br>**DM1=**76.0 (73.5-84.4) vs **Controls=** 85.5 (79.2-98.0); p=Ns	Median (interquartile):<br>Total-Cholesterol<br>**DM1=**208(181–227) vs **Controls=**200(180–224); p=Ns;<br>TAG<br>**DM1=**129(95-189) vs **Controls=**92(74-125); p=0.005<br>HDL-Cholesterol<br>**DM1=**48(39–57) vs **Controls=**47(40–54); p=Ns;<br>LDL-Cholesterol<br>**DM1=**129(92-148) vs **Controls=**131(107-156), p=Ns<br>Diabetes mellitus, %<br>**DM1=**1/32 vs **Controls=**0/32 p=0.04
Ben Hamou, A.,et al. [28], France, 2019	**DM1 (n):**115<br>Sex: 71F, 44M<br>Age:45.1y	CTG (media (IQR))= 500(260-850)<br>BMI (kg/m²)<br>**DM1=**26.4±6.5	**DM1=**51.38±42.36	ND	**DM1=**90.0 ± 15.0	Total cholesterol<br>**DM1=**200.0 ± 40.0<br>TAG (median (IQR))<br>**DM1=**132 (97–192)<br>LDL (median (IQR))<br>**DM1=**117 (96–140)<br>HDL (median (IQR))<br>**DM1=**50 (43–59)<br>Diabetes (n/N; %)<br>**DM1=**30/115 (26.1);
Vujnic, M., et al [63], Bosnia and Herzegovina, 2015	**DM1 (n):** 66<br>Sex: 33F, 33M<br>Age: 41.9y	CTG=751.9±280.6<br>BMI (kg/m²)<br>**DM1=**23.1±4.5	ND	ND	**DM1=**88.28±16.21	Total cholesterol<br>**DM1=**228.15± 54.14<br>TAG<br>**DM1=**194.86± 124<br>LDL<br>**DM1=**143.08±50.27<br>HDL<br>**DM1=**50.27± 11.6<br>Low HDL (%)<br>**DM1=**34.8<br>Metabolic syndrome (n, %)<br>**DM1=** 11,16.7<br>Central obesity- (n, %):<br>**DM1=**9,13.6
Renna, L.V., et al. [70], Italy, 2019	**DM1 (n):** 8<br>Sex: 6F, 2M<br>Age: 34y<br>**Control (n):** 3<br>Sex: 2F, 1M<br>Age: 43y	CTG=413,75 (230-800)<br>BMI (kg/m²)<br>**DM1=**23.3;<br>**Control=**23.7	ND	**DM1=**2.25;<br>**Control=**1.6	ND	ND

Renna, L.V., et al. [55], Italy, 2017	**DM1 (n):** 8<br>Sex: 7F, 1M<br>Age: 38y<br>**Control (n):** 8<br>Sex: 4F, 4M<br>Age: 35y	CTG=370,6±111.1 (220-560)<br>BMI (kg/m^2)<br>**DM1=**23.6<br>**Control=**22.7	**DM1=**65.62	**DM1=**1.945	**DM1=**82.75;<br>**Control=**89.375	Total Cholesterol<br>**DM1=**176;<br>**Control=**146
Stojanovic, R.V., et al. [56], Serbia, 2010	**DM1 (n):** 34<br>Sex: 18F, 16M<br>Age: 43y<br>**Control (n):** 34<br>Sex: 18F, 16M<br>Age: 43y	CTG: ND<br>BMI (kg/m^2)<br>**DM1=**23.5±4.3 (15.6-30.1); 23±4.4F; 24.1±4.2M;<br>**Control=** 23.5±4.	**DM1=**135.48±74.30F; 151.45±59.16M; p= Ns	**DM1=**3.78±2.03F; 4.77±2.81M	**DM1=**78.91±8.28F; 86.12±20.18M	Total Cholesterol<br>**DM1=**210.75±55.68F; 251.74±62.65M<br>TAG<br>**DM1=**52.98±19.335F; 88.55±37.9M
Daniele, A., et al [57], Italy, 2011	**DM1 (n):** 21<br>Sex: 8F, 13M<br>Age: 44.5y<br>**Control (n):** 82<br>Sex: 43F, 39M<br>Age: 39.2y	CTG=ND<br>BMI (kg/m^2)<br>**DM1=**25.7±3.6, Median=25.2<br>**Control=**23.2±2.9, Median=23.4; p=0.001<br>Waist circumference(cm)<br>**DM1=**97.4 (8.6); Median=95<br>**Controls=**84.9 (11.6); Median=89; p<0.001	**DM1=**95.83±63.19, Median=75 vs **Control=**45.83±12.50, Median=41.6; p<0.001	**DM1=**3.6±2.2, Median=3.0 vs **Control=**1.3±0.2Median=1.3; p<0.001	**DM1=**108.5±42.2, Median=100.0 vs **Control=**81.6±11, Median=80.0; p<0.001	Total cholesterol<br>**DM1=**210.8±51.3; median=198.5 vs **Control=**194.5±38.9; median=190.0; p<0.001<br>TAG<br>**DM1=**244.1±186, median=146 vs **Control=**95.2±53.9 Median=82.5; p<0.001
Shieh, K., et al. [58], USA, 2010	**DM1 (n):** 36<br>Sex: 20F, 16M<br>Age: 42.2y	CTG=ND<br>BMI (kg/m^2)<br>**DM1=**27.2(19-38)<br>Waist circumference (cm)<br>**DM1=**94.3(71 – 119)	**DM1=**186.11 (13.8–1229.16)	**DM1=**6.4 (0.4–35.0)	**DM1=**97.0 (78–138)	Total cholesterol<br>**DM1=**195.2(129-286)<br>TAG<br>**DM1=**162.8(29–320)<br>LDL cholesterol<br>**DM1=**111.2(59–194)<br>HDL cholesterol<br>**DM1=**51.4(28–93)<br>Diabetes (n, %)<br>**DM1=**4, 11.8<br>Metabolic syndrome (%)<br>**DM1=**14 (41.2%)
Johansson, A., et al. [59], Sweden, 2001	**DM1 (n):** 42<br>Sex: 20F, 22M<br>Age (median): 41.5y (IQR:28.5-58.7)<br>**Control (n):** 50<br>Sex: 23F, 27M<br>Age (median): 42y (IQR: 27.0-56.9)	Median (10th and 90th percentil)<br>CTG=679(152-1142)<br>BMI (kg/m^2)<br>**DM1=**23.3(18.6-29.2) vs **Control=**24.0 (20.7-30.0); p=Ns<br>Waist circumference (cm)<br>DM1=89.3(69.1–108.9) vs Controls=82.5(70.0–103.0); p=Ns	Median (10th and 90th percentil)<br>**DM1=**64.58(33.33–155.55) vs **Control=**40.27(20.83–75.69); p<0.001	ND	ND	Median (10th and 90th percentil)<br>Total cholesterol<br>**DM1=**208.8(139.2–278.4) vs **Control=**193.35; (158.55–239.75); p= Ns<br>TAG<br>**DM1=**168.3(88.57–310) vs **Control=**83.26(52.26–150.58) p<0.001<br>LDL cholesterol<br>**DM1=**127.6(69.6–185.6) vs **Control=**127.6 (85.07–166.28) p=Ns<br>HDL cholesterol<br>**DM1=**47.56(34.38–66.13) vs **Control=**51.04(40.6–76.18) p <0.05

Perseghin, G., et al. [64], Italy, 2004	**DM1 (n):** 10<br>Sex: 8F, 2M<br>Age: 38y<br>**Control (n):** 10<br>Sex: 8F, 2M<br>Age: 33y	CTG= ND<br>BMI (kg/m^2)<br>**DM1**=22.3±1.4<br>**Control**=21.7±1.2	ND	ND	**DM1**=82.70±4.14 vs<br>**Control**=91.71±2.34; p<0.05	Total cholesterol<br>**DM1**=186±11.21 vs<br>**Control**=176.72±11.21; p=Ns<br>TAG<br>**DM1**=108.06±15.94 vs<br>**Control**=77.06±28.3; p=Ns<br>LDL cholesterol<br>**DM1**=106.34±13.14 vs<br>**Control**=97.45±8.50; p=Ns<br>HDL cholesterol<br>**DM1**=58.39±3.09 vs<br>**Control**=51.82±2.32; p=Ns
Spaziani, M., et al. [60], Italy, 2020	**DM1 (n):** 63<br>Sex: 27F, 36M<br>Age: 43y<br>**Control (n):** 100<br>Sex: All male<br>Age (mean): 42	CTG Range= 50 to >1000	**Male**<br>**DM1**=104.16±97.22<br>**Controls**=56.25±43.75; p=Ns<br><br>**Female**<br>**DM1**=104.16±76.38<br>**Controls**=63.19±18.75; p=Ns	ND	**Male:**<br>**DM1**=95±17 vs<br>**Control**=83±7.9;<br>p< 0.005;<br><br>**Female:**<br>**DM1**=113±63 vs<br>**Controls**=88±9.0;<br>p< 0.0001	**Male**<br>Total cholesterol<br>**DM1**=181±43 vs **Control**=128 (20) p< 0.0001;<br>TAG<br>**DM1**=186± 87 vs **Controls**=75 (32); p< 0.0001<br>LDL cholesterol<br>**DM1**=104±29 vs **Control**=46 (12); p< 0.0001<br>HDL cholesterol<br>**DM1**=44±9.1 vs **Control**=67 (19) p< 0.0001;<br><br>**Female:**<br>Total cholesterol<br>**DM1**=191±27 vs **Controls**=134 (20); p< 0.0001<br>TAG<br>**DM1**=147±82 vs **Controls**=105 (20); p< 0.05<br>HDL cholesterol<br>**DM1**=61±21 vs **Controls**=55 (8.9); p=NS<br>LDL cholesterol<br>**DM1**=101±25 vs **Controls**=102 (21); p=NS
Johansson, A., et al. [61], Sweden, 2002	**DM1 (n):** 18<br>Sex: all male<br>Age (median): 39y (percentil: 22-90)<br>**Control (n):** 18<br>Sex: all male<br>Age (median): 38y, (percentil 23-62)	Median 10th-90th percentiles<br>CTG=614.5 (347-1088)<br>BMI (Kg/m^2)<br>**DM1**=24.7(18.9-30.3) vs **Control**=24.6(19.8-30.7); p=Ns	Median 10th-90th percentiles<br>**DM1**=83.0(55.6–210.4) vs<br>**Control**=51.0(34.0–106.7);<br>p<0.01	Median 10th-90th percentiles<br>**DM1**=2.3(1.4–5.5) vs<br>**Control**=1.4(0.90–3.5);<br>p<0.001	Median 10th-90th percentiles<br>**DM1**=82.88(72.07–95.49) vs<br>**Control**= 79.28 (68.46–91.89);<br>p=Ns	ND
Hudson, A.J., et al. [62], England, 1987	**DM1 (n):** 10,<br>Sex: 6F; 4M<br>Age: 45y	CTG=ND	**DM1**=181.1±25.5 vs<br>**Control**=86.0±3.7;	ND	**DM1**=93.69±1.8 vs<br>**Control**=90.09±1.80; p=Ns	**Mean (5th and 95th percentil)**<br>Total cholesterol<br>**DM1**=210(183-237)

	**Control (n):** 10; Sex: 6F; 4M<br>Age: 45 y		p<0.001			<u>TAG</u><br>**DM1=**274(127-421)<br><u>VLDL</u><br>**DM1=**180(32-328)<br><u>LDL cholesterol</u><br>**DM1=**120(98-143)<br><u>HDL cholesterol</u><br>**DM1=**57(46-68)<br><u>LDL apolipoprotein B</u><br>**DM1=**102.0+ 8.4 vs **Control=** 84.3 ±4.0; p<0.05
Moorjani, S., et al. [68], Canada, 1989	**DM1 (n):** 70<br>Sex: 35F, 35M<br>Age: 36 y<br>**Control (n):** 81<br>Sex: 50F, 31M<br>Age: 39y	<u>CTG=</u>ND	ND	ND	ND	<u>TAG</u><br>**DM1=** 216± 17 vs **Controls** =168 ±9; p<0.01<br><u>VLDL cholesterol</u><br>**DM1=** 32.4±4.0 vs **Controls=** 21.0±1.3; p< 0.01<br><u>LDL cholesterol</u><br>**DM1=** 111.3±4.4t vs Controls= 122.6 ±3.8;<br>p< 0.05<br><u>HDL cholesterol</u><br>**DM1=** 48.2 ±1.4 vs **Controls=** 45.7 ±1.3; p=Ns
Takada, H., et al [69], Japan, 2016	**DM1 (n):** 91<br>Sex: 41F, 50M<br>Age (median): 47y	<u>CTG (Median)=</u>1075	ND	ND	ND	<u>TAG</u> (abnormality) 38 cases (42%)<br>**DM1 (median):** 129 mg/dL<br><u>LDL cholesterol</u> (abnormality) 19 cases (21%) **DM1 (median):** 117 mg/dL<br><u>HDL cholesterol</u> (abnormality) 14 cases (15%)<br>**DM1 (median):** 51
Matsumura, T., et al. [65], Japan, 2009	**DM1 (n):** 95<br>Sex: 45F, 50M<br>Age: 43y<br>**Control (n):** 734<br>Sex: 193F, 541M<br>Age: 54.6±8.5	<u>CTG=</u> 973±744<br><u>BMI (Kg/m^2)</u><br>**DM1:** 21.4±4.9<br>**Control:** 23.2±2.8	<u>Insulinogenic index</u><br>**DM1:** 1.71±0.29 vs<br>**Control:** 0.77±0.12; p= 0.004	**DM1:** 1.96± 0.11 vs<br>**Control:** 1.36±0.04; p <0.001	**DM1:** 94.2±1.7 vs<br>**Control:** 95.6± 0.6; p=0.455	<u>Total cholesterol</u><br>**DM1:** 208.7±3.7 vs<br>**Control:** 200.5±1.2; p= 0.038<br><u>TAG</u><br>**DM1:** 172.4±91 vs<br>**Control:** 123.4±3.0; p < 0.001<br><u>HDL</u><br>**DM1:** 52.2±2.0 vs<br>**Control:** 56.7±0.6; p= 0.035
Heatwole, R.C., et al. [66], USA, 2011	**DM1 (n):** 15<br>Sex: 8F, 7M<br>Age: 42.7±10.4	<u>CTG:</u> 355.9±209.6<br><u>BMI (Kg/m^2):</u> 23.0±3.9			**DM1:** 88.57±10.12	<u>TAG</u><br>**DM1:** 140.27± 60.65<br><u>HDL</u><br>**DM1:** 49.93±15.64
Perna, A., et al., [67], Italy, 2020	**DM1 (n):** 61<br>Sex: 26F, 35M<br>Age: 47.2±13.8	<u>CTG (n=53):</u> 466.17±269.32<br><u>BMI (Kg/m^2):</u> 24.21±4.78<br><u>BMI (Kg/m^2) ≥ 30:</u> n= 8			**DM1:** 91.8±26.69	<u>Total cholesterol</u><br>**DM1 (n=59):** 188.22±40.62<br><u>TAG</u><br>**DM1 (n=59):** 152.41±71.14<br><u>Diabetes type II:</u><br>**DM1:** n= 6<br><u>Hypercholesterolemia (>200 md/dL)</u><br>**DM1:** n= 21

Data are presented as mean or mean±standard deviation (range) or [IQR], unless otherwise stated; Abbreviations: Ns- Not significant; F- Female; M-Male; BMI- Body Mass Index; HDL- High-density lipoprotein; LDL- Low-density lipoprotein; TAG- Triacylglycerol; ND- Not Determined.

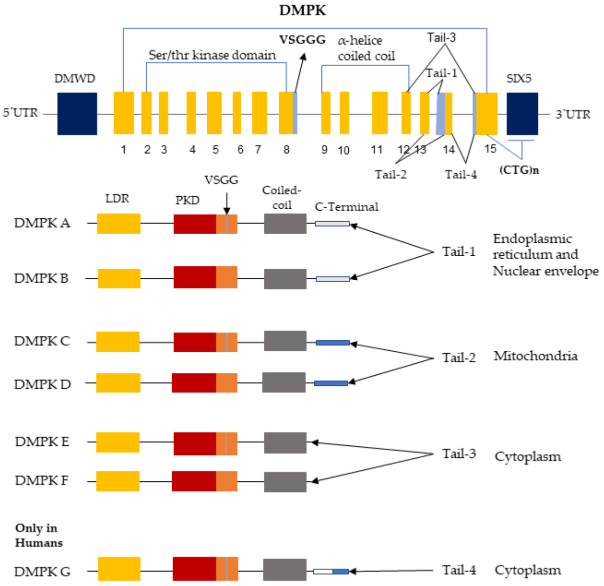

Figure S1. Schematic representation of DM1 gene and isoforms. (A) DMPK gene and major DMPK isoforms in humans and their domains and localization [5,6,7]. DMPK subcellular localization is confined to either endoplasmic reticulum (ER) or nuclear envelope (DMPK A and B), to mitochondria (DMPK C and D) or to cytoplasm (DMPK E, F, and G). [6,36,20]. UTR, untranslated region; DMWD, Dystrophia myotonica WD repeat-containing protein (represented as Blue); DMPK, Myotonic Dystrophy Protein Kinase; CTG, cytosine-thymine-guanine trinucleotide; LDR-Leucine rich Domain; PKD, Serine/threonine protein kinase domain. Alternative splicing sites of VSGGG and Tail (1, 2, 3, and 4) are represented as light blue.
